# Molecular insights of exercise therapy in disease prevention and treatment

**DOI:** 10.1038/s41392-024-01841-0

**Published:** 2024-05-29

**Authors:** David Walzik, Tiffany Y. Wences Chirino, Philipp Zimmer, Niklas Joisten

**Affiliations:** 1 Division of Performance and Health (Sports Medicine), Institute for Sport and Sport Science, TU Dortmund University, 44227 Dortmund, North Rhine-Westphalia Germany; 2 Division of Exercise and Movement Science, Institute for Sport Science, University of Göttingen, 37075 Göttingen, Lower Saxony Germany

**Keywords:** Molecular medicine, Molecular biology, Cell biology, Physiology

## Abstract

Despite substantial evidence emphasizing the pleiotropic benefits of exercise for the prevention and treatment of various diseases, the underlying biological mechanisms have not been fully elucidated. Several exercise benefits have been attributed to signaling molecules that are released in response to exercise by different tissues such as skeletal muscle, cardiac muscle, adipose, and liver tissue. These signaling molecules, which are collectively termed *exerkines*, form a heterogenous group of bioactive substances, mediating inter-organ crosstalk as well as structural and functional tissue adaption. Numerous scientific endeavors have focused on identifying and characterizing new biological mediators with such properties. Additionally, some investigations have focused on the molecular targets of exerkines and the cellular signaling cascades that trigger adaption processes. A detailed understanding of the tissue-specific downstream effects of exerkines is crucial to harness the health-related benefits mediated by exercise and improve targeted exercise programs in health and disease. Herein, we review the current in vivo evidence on exerkine-induced signal transduction across multiple target tissues and highlight the preventive and therapeutic value of exerkine signaling in various diseases. By emphasizing different aspects of exerkine research, we provide a comprehensive overview of (i) the molecular underpinnings of exerkine secretion, (ii) the receptor-dependent and receptor-independent signaling cascades mediating tissue adaption, and (iii) the clinical implications of these mechanisms in disease prevention and treatment.

## Introduction

Physical inactivity is associated with the development of various chronic diseases including cancer, cardiovascular, metabolic, and neurodegenerative diseases.^1,2^ In contrast, physical activity can prevent these diseases^3^ and is therefore recommended as a measure to improve public health and reduce disease burden.^4^ In this context, exercise training—defined as the planned and structured, recurrence of acute exercise bouts with the aim to maintain or increase physical aptitude^5^—is a low-cost lifestyle intervention that can ameliorate and prevent numerous pathological conditions.^6–8^ During acute exercise, multiple physiological parameters (e.g., respiration, heart rate, hormone secretion) are regulated to cover the increased demand for oxygen and nutrients of metabolically active tissues such as cardiac and skeletal muscle.^9^ Systematical exposure to recurring exercise stimuli results in long-term adaptions of various tissues and induces a myriad of well-known exercise effects, such as increased vascularization and mitochondrial biogenesis, improved cardiac and immune cell function, and enhanced substrate handling by adipose and liver tissue.^10^ Ultimately, these adaptions result in a pre-conditioned state that protects trained individuals from future (patho)physiological challenges such as exercise or (chronic) disease.^11^ Despite the substantial health-related benefits of exercise training, the precise molecular signaling processes leading to structural and functional tissue adaption remain largely unknown.

Overcoming these uncertainties, several exercise-inducible signaling molecules have been discovered. During acute exercise, biological compounds with autocrine, paracrine and/or endocrine function are secreted by different tissues, including but not limited to skeletal and cardiac muscle, liver, and adipose tissue. These compounds, which were collectively termed *exerkines*,^12,13^ form a heterogeneous group of signaling molecules, comprising peptides and proteins, tissue metabolites, lipids, and nucleic acids, some of which also function as hormones or cytokines.^14–16^ As reviewed by Li and colleagues,^17^ uncovering the global dynamics of exerkine activity is crucial to understand the physiological effects of exercise on the human organism—especially to justify exercise prescription for the prevention and treatment of chronic diseases. This understanding includes mechanistic knowledge on *exerkine kinetics*, i.e., the secretion, distribution, metabolization, and elimination of exerkines as well as *exerkine dynamics*, i.e., the receptor-dependent or receptor-independent interaction of exerkines with target cells, dose-response relationships, exerkine-induced signaling pathways, and downstream adaption processes. Transferring these mechanistic insights into different clinical settings has shown promising implications in disease prevention,^18–21^ exercise therapy,^22–24^ and the development of pharmaceutical exercise mimetics.^10,25^

In this review, we provide a comprehensive overview of the tissue-wide health effects mediated by exercise, with a special focus on cellular mechanisms governing the preventive and therapeutic impact of exerkines across different target cells. After summarizing identified exerkines together with their source tissues, their mode of secretion, and their local or systemic distribution (“Exerkines: exercise-inducible signaling molecules”), we highlight receptor-dependent and receptor-independent signaling pathways that converge in biological adaption processes of exerkine-stimulated target cells (“Exerkine-induced signal transduction and biological tissue adaption”). Leveraging these mechanistic insights, we conclude with the clinical relevance of these findings and discuss the current knowledge and progress of exerkine signaling in different disease settings (“Exercise therapy in disease prevention and treatment”). A profound understanding of the molecular foundation of tissue-wide exercise-mediated health effects is crucial to harness the preventive and therapeutic potential of exercise, develop tailored exercise programs, and consolidate the role of exercise therapy in clinical routine.

## Exerkines: exercise-inducible signaling molecules

The communicative interconnection of different cells allows mammals to adjust their physiology to environmental cues such as nutrient uptake (or scarcity), changes in temperature, or fight-or-flight encounters. Unsurprisingly, the onset of exercise is characterized by profound alterations in physiology and increased metabolic communication across multiple organ systems. This inter-organ crosstalk involves both, signaling molecules with immediate effects such as hormones, and signaling molecules that have longer-term effects by inducing structural and functional tissue adaption. In this review, we will focus on the latter, i.e., exercise-mobilized signaling molecules (exerkines) that induce adaptive processes in target cells and tissues. Of note, many exerkines are altered by a single exercise session, while some only change in response to exercise training (i.e., repeated exercise bouts). The effect of these exerkines on adaption processes might differ between acute and chronic exercise settings.^26^

### Molecular diversity of exerkines

Exerkines differ in their molecular structure, ranging from peptides and proteins over tissue metabolites and lipids to nucleic acids.^14,27–29^ Despite this chemical distinction being unambiguous, there are several other ways of classifying exerkines, some of which are even more intuitive. For instance, many exerkines can be classified as hormones, cytokines, or chemokines, and hybrid molecules such as peptide hormones (e.g., irisin)^30^ or glycoproteins (e.g., follistatin-like 1)^31–33^ exist. These redundant ways of classifying exerkines highlight that a distinction based on chemical structure—as we have chosen here—or physiological function might be more suitable, depending on the scientific focus.

#### Peptides and proteins

Of the different classes of exerkines known today, peptides and proteins lie at the center of intercellular communication. Since the proteinogenic makeup of different cells defines their morphological and functional characteristics—e.g., protein and/or enzyme abundance varies considerably between cell types and thus equips these cells with different functions—it is unsurprising that a protein-based communication system has evolved to share information and facilitate crosstalk between cells.^34^ Human trials have repeatedly identified hundreds of proteins mobilized into the bloodstream in response to acute exercise^35–37^ and exercise training.^38,39^ These investigations have led to the finding that acute exercise and exercise training are accompanied by profound intercellular communication via secreted peptides and proteins. Although this depicts a crucial step towards a comprehensive molecular understanding of the positive health effects of exercise, few investigations have characterized these exerkines in a more holistic manner, i.e., with respect to the source tissues, the local or systemic distribution, the tissue-specific signaling cascades and biological adaption processes.

In this context, a recently published study used state-of-the-art technology to identify over 200 cell type-specific proteins secreted into the blood in response to exercise training in mice.^40^ Besides describing source tissues for many exerkines, this study also elucidated the mode of secretion of a novel proteinogenic exerkine secreted from the liver, and established a mechanistic link to anti-obesity, anti-diabetic, and endurance-enhancing effects in mice.^40^ This demonstrates how both explorative proteomics-based approaches as well as mechanistic hypothesis-driven experiments aid in unraveling the molecular mechanisms by which proteins are mobilized in response to acute exercise and exercise training. Apart from this example, there are numerous further exercise-secreted proteins whose molecular underpinnings have been characterized to different extents (Table 1).

#### Tissue metabolites and lipids

Similar to peptides and proteins, tissue metabolites and lipids are increasingly recognized to possess signaling properties as well.^28^ In exercise context, a well-known metabolite with such properties is lactate. Despite its initial perception as a waste product of glycolysis with detrimental effects to muscle physiology, lactate is nowadays viewed as a metabolic intermediate that is secreted into circulation from tissues with high energy turnover (e.g., skeletal muscle tissue).^41–43^ Additionally, lactate has numerous effects on target cells via receptor-dependent and receptor-independent mechanisms.^41,44,45^ More recently, exercise-mobilized lactate was additionally found to undergo a condensation reaction with the essential amino acid phenylalanine in carnosine dipeptidase 2 positive cells such as monocytes, macrophages, and epithelial cells, yielding N-lactoyl-phenylalanine (Lac-Phe), a signaling metabolite that suppresses feeding and obesity via so far unknown mechanisms.^17^ This exemplifies how metabolic exerkines can interact with other biomolecules, thereby adding further complexity to the effects of exerkines on distinct target cells.

Furthermore, tricarboxylic acid (TCA) cycle intermediates have received much attention as exercise-responsive signaling molecules^46,47^ due to their central role in intermediary metabolism.^48,49^ For instance, plasma levels of succinate increase substantially in response to acute exercise^50^ and paracrine signaling from skeletal muscle tissue to non-myofibrillar muscle-resident cell types such as immune and endothelial cells was shown to confer skeletal muscle and extracellular matrix remodeling.^51–53^ In view of the endocrine mobilization of succinate, adaptions in other, more distant target tissues were also described.^54,55^

Apart of these examples, exercise is accompanied by alterations in numerous further metabolites, including lipids such as 12,13-dihydroxy-9Z-octadecenoic acid (12,13-diHOME),^56^ and the nonprotein amino acid β-aminoisobutyric acid (L-BAIBA).^57,58^ Additionally, plasma metabolomics approaches are increasingly shedding light at the wide array of metabolites mobilized in response to an acute bout of exercise.^35,47,50^ As evidenced by time-resolved plasma metabolome profiling in response to endurance and resistance exercise, these metabolites differ in dependence on the applied exercise modality.^59^ Since the signaling properties of these exercise-responsive metabolites are only recently moving into scientific focus,^28^ future research will have to show how these different metabolites participate in inter-organ crosstalk and tissue adaptions.

#### Nucleic acids

A further class of biomolecules with signaling properties in the context of exercise are non-coding RNAs. The principal characteristic of these nucleic acids is that they are not translated into proteins. Non-coding RNAs include microRNAs (miRNA), circular RNAs, and long non-coding RNAs (lncRNA), all of which interact with numerous cellular processes such as transcription, post-transcriptional regulation, genome integrity, and organelle function via distinct mechanisms.^60,61^ Importantly, non-coding RNAs are cell type-specific^62,63^ but can also exert their functions in other cell types via paracrine and/or endocrine signaling through extracellular vesicles (EVs).^64–66^

In clinical context, miRNAs (typically 18–25 nucleic acids in length) are increasingly attracting attention due to their predictive and prognostic value as biomarkers in different disease settings. For instance, miR-210 and miR-222 are useful biomarkers of future cardiovascular disease, as they are associated to low V̇O_2max_ levels.^67^ Additionally, miR-210 is associated with mortality in patients with acute dyspnea,^68^ and serves as prognostic marker in moderate to severe aortic stenosis.^69^ Other miRNAs such as miR-106a-5p, miR-424-5p, let-7g-5p, miR-144-3p and miR-660-5p were shown to predict future risk of fatal acute myocardial infarction in healthy individuals.^70^ In post-myocardial infarction heart failure, preclinical experiments have identified miR-214-3p, miR-497-5p, and miR-31a-5p as potential therapeutic targets, as they contribute to heart failure-like behavior in calcium handling and electrophysiology in response to exercise training.^71^ Similarly, miR-210 was shown to increase in response to exercise training in the heart and blood of rodents and after cardiac rehabilitation in patients with coronary heart disease. Underlining the therapeutic potential of miR-210, it was shown to promote cardiomyocyte proliferation and survival and contribute to cardiac protection against ischemia/reperfusion injury in mice.^72^ Further exercise-mobilized miRNAs, such as miR-143, miR-338, mir-155, miR-181a, miR-30a, and miR-142^73^ mediate many of the well-known exercise effects in both, the innate and adaptive immune system, favoring processes such as T cell differentiation^74^ and activation,^75^ and an anti-inflammatory phenotype switch in adipose tissue macrophages.^76^ Exercise training additionally regulates miRNAs in tissues including skeletal muscle, cardiac muscle, and nervous tissue.^77^ In skeletal muscle, miRNAs induce post-transcriptional regulation of genes involved in muscle regeneration and mitochondrial biogenesis.^78,79^ A comprehensive overview of miRNAs involved in exercise adaption was given by Silva and colleagues.^77^

Exercise also increases lncRNAs (typically >200 nucleic acids in length) in skeletal muscle tissue.^80,81^ Prime examples for this are taurine-upregulated gene 1, a lncRNA that serves as transcriptomic regulator in skeletal muscle adaption to exercise and induces differential expression of hundreds of genes in vitro,^82^ and lncRNA CYTOR, which regulates fast-twitch myogenesis, muscle mass, and fitness in ageing.^81^ Beside these implications for skeletal muscle tissue, lncRNAs were also found to signal between different tissues via EVs. For instance, colorectal neoplasia differentially expressed (CRNDE), a lncRNA with high abundance in exercise training-derived EVs of mice, was shown to protect cardiomyocytes from hypoxia/reoxygenation damage as it occurs during myocardial infraction.^83^ Mechanistically, both CRNDE and miR-489-3p were shown to participate in this protective effect of exercise on cardiac muscle tissue,^83,84^ highlighting the dynamic interconnection of lncRNAs and miRNAs in the regulation of transcriptomic programs in response to exercise. Similar cardioprotective effects were also found for the lncRNAs cardiac physiological hypertrophy-associated regulator (CPhar) and lncExACT1. While CPhar was increased with exercise training and triggered physiological cardiac hypertrophy,^85^ lncExACT1 decreased in response to exercise training and alleviated pathological hypertrophy in mice.^86^ This demonstrates how different lncRNAs are involved in cardiac remodeling in response to exercise training and how exercise fine-tunes these molecular processes to enable cardiac adaption. Understanding the mechanisms by which lncRNAs induce exercise adaptions can also inspire novel therapeutic approaches based on the health-promoting effects of exercise.

Besides lncRNAs, exercise-induced circular RNAs like circUtrn and circ-Ddx60 were recently shown to mediate cardioprotective effects as well.^87,88^ Of interest, in a mouse model of pathological cardiac hypertrophy, circ-Ddx60 was crucially involved in an antihypertrophic response of cardiac muscle tissue, which occurred after exercise hypertrophic preconditioning.^87^ This suggests a cardiac antihypertrophic memory after previous exercise training. From a translational perspective, this demonstrates that exercise trained cardiac muscle tissue might by protected from future pathological hypertrophy via the action of circ-Ddx60. However, to prove this, further research in different patient collectives is needed.

Concerning the molecular regulation of exercise programs via non-coding RNAs, a further level of complexity is added by competing endogenous RNAs (ceRNAs), which comprise protein-coding ceRNAs, pseudogenes, lncRNAs, and circular RNAs. ceRNAs share miRNA response elements with mRNA molecules, and thus compete for the same miRNAs, thereby regulating transcription.^89,90^ The interaction of different ceRNAs has led to the analysis of ceRNA networks which have also been applied in exercise context to improve our understanding of the complex transcriptional programs of skeletal muscle tissue in response to exercise training.^91,92^ Additionally, epigenetic modification of mRNA was shown to participate in tissue adaption to exercise training. In detail, exercise training reduced N^6^-methyladenosine modification (m^6^A) of RNA in cardiac muscle tissue via the action of m^6^A methyltransferase 14 (METTL14), thereby alleviating ischemia/reperfusion injury and cardiac dysfunction during cardiac remodeling in mice.^93^ A similar dependency on m^6^A of RNA was also reported for anxiolytic effects of exercise training mediated by epigenetic modification of the medial prefrontal cortex of mice. Interestingly, these effects were dependent on hepatic biosynthesis of the methyl donor S-adenosyl methionine, thus providing initial evidence for a liver-brain axis that participates in the exercise-induced prevention of anxiety via epigenetic modification of RNA.^94^

### Source tissues and exerkine secretion

#### Molecular triggers of exerkine secretion

For any given secretion of peptides, proteins, tissue metabolites, or nucleic acids in response to exercise, a molecular trigger causing the release from their source tissue is required. For instance, in skeletal muscle tissues of mice the production and secretion of the exerkine musclin, is mediated by the calcium-dependent activation of AKT1,^95^ while vascular endothelial growth factor (VEGF) is secreted in response to hypoxia,^96^ and succinate secretion was shown to depend on changes in intracellular pH.^52^ Some exerkines are also subject to hormonal regulation. For instance angiopoietin-like 4 (ANGPTL4) is regulated by glucagon-cAMP-PKA signaling, thus expanding the molecular triggers of exerkine secretion by hormonal effects.^97^ Moreover, exercise-dependent shear stress on endothelial cells was also shown to trigger exerkine secretion.^98^

#### Secretion of peptides and proteins

Building upon these molecular triggers, the mode of secretion can also vary in dependence on the molecular structure of different exerkines. Secretory peptides and proteins usually contain a signal peptide, that is cleaved during maturation of the protein along the ER-Golgi secretory pathway.^99,100^ Some proteinogenic exerkines such as apelin are additionally translated as propeptides and can be cleaved at different locations in dependence on proteolytic processing of the peptide precursor (e.g., apelin-36 vs. apelin-13). Of note, the receptor affinity of these peptides differs,^101,102^ which might impact the durability of apelin receptor signaling in the context of exercise. Once transported to the cell membrane, there are two fates for proteins: either the protein remains in the plasma membrane, or it is secreted into the extracellular space.^99,100^ For instance, fibronectin type III domain containing 5 (FNDC5) is a type I transmembrane protein^100^ containing a 31 amino acid signal peptide and a 112 amino acid polypeptide that is secreted into the extracellular space after proteolytic cleavage from FNDC5.^30^ In analogy to the Greek messenger goddess Iris, this secreted polypeptide was named irisin due to its signaling function from skeletal muscle to distinct target tissues in response to exercise. In contrast, many other proteinogenic exerkines are assumed to be secreted from their source tissues directly, although secretion pathways can differ^103^ and unconventional protein secretion—particularly in form of EVs—might also play a role in the context of exercise.^12,13,104,105^

#### Secretion of tissue metabolites and lipids

In contrast to peptides and proteins, secretion of tissue metabolites is governed by mass action. Metabolic flux, enzyme abundance and activity, and presence of metabolite-specific transporters are crucial characteristics that impact the secretion of metabolites in response to exercise. A well-investigated exercise-responsive metabolite that exemplifies these molecular underpinnings is lactate. With the onset of exercise, glycolytic flux of skeletal muscle tissue rises, yielding pyruvate as an end-product, which is imported into mitochondria in a process dependent on mitochondrial pyruvate carriers (MPCs). A fraction of cytosolically accumulating pyruvate is additionally shunted towards lactate via lactate dehydrogenase (LDH). During low- and moderate-intensity exercise cytosolic accumulation of pyruvate is alleviated by mitochondrial import via MPCs, however, at higher intensities this import becomes saturated, creating a metabolic bottleneck and thus favoring the formation of lactate via LDH. Thus, although both pyruvate and lactate concentrations increase in response to exercise, lactate production outperforms mitochondrial pyruvate import at higher work rates, as indicated by rising lactate/pyruvate ratios.^106,107^ In consequence of these molecular events, rising intracellular lactate levels create a concentration gradient across the cell membrane which is alleviated through excretion and systemic deployment of lactate, a well-documented hallmark of acute exercise. Due to locally altered metabolism, skeletal muscle tissue is thus perceived as a lactate-producing tissue in the context of exercise, thereby supplying other tissues with a three-carbon energy source and signaling metabolite.^43^

A further exerkine that exemplifies the molecular underpinnings of metabolite secretion is succinate. In search of a molecular explanation for the preferential mobilization of succinate over other TCA metabolites,^50^ Reddy and colleagues revealed that contraction-induced acidification of skeletal muscle tissue preferably protonates succinate—a reaction that appears reasonable, given that succinate has the highest p*K*_a2_ (dissociation constant between dicarboxylate and monocarboxylate) of all TCA metabolites.^52^ Under physiologically acidic conditions found in skeletal muscle tissue during exercise (pH ~ 6.4–6.8), succinate is protonated from a dicarboxylate to a monocarboxylate, rendering it an available substrate for transport across the cell membrane via monocarboxylate transporter 1. This introduces a pH-gated secretion mechanism for succinate that explains its preferential mobilization from skeletal muscle tissue in response to acute exercise.^52^

Collectively these examples highlight how metabolic flux, enzyme abundance and activity, intracellular pH, and membrane transport systems for specific metabolites define the ability of tissue metabolites to act as local or systemic exerkines. Similar mechanisms have not been investigated in such detail for other metabolic exerkines such as kynurenic acid (KYNA), SPARC, and L-BAIBA or for exercise-responsive lipids like 12,13-diHOME (Table 1).

**Table 1:** Overview of exercise-inducible singling molecules (exerkines)

Exerkine	Described source tissues	Mode of intercellular communication			Refs
		Autocrine	Paracrine	Endocrine	
Peptides and proteins					
ANGPT1	Skeletal muscle	✓	✓	✓	^214^
FGF21*	Skeletal muscle	×	×	✓	^190,408^
GDF15*	Skeletal muscle	✓	✓	✓	^409^
IL-6*	Skeletal muscle	✓	✓	✓	^410,411^
IL-7*	Skeletal muscle	✓	✓	×	^412^
IL-8*	Skeletal muscle endothelium	✓	✓	×	^230,413^
IL-15*	Skeletal muscle	✓	✓	✓	^144,162^
Musclin	Skeletal muscle	✓	✓	×	^414^
Myonectin*	Skeletal muscle	×	×	✓	^134,415^
NTN	Skeletal muscle	✓	✓	×	^146^
SPARC*	Skeletal muscle	✓	✓	×	^130,131^
VEGF*	Skeletal muscle	✓	✓	✓	^416–420^
FN1	Skeletal muscle	×	×	✓	^250^
FST*	Skeletal and cardiac muscle, hepatic	×	×	✓	^239,421–423^
FSTL1*	Adipose, skeletal muscle, cardiac muscle	✓	✓	✓	^31,32,424,425^
Fractalkine*	Skeletal muscle, endothelium	×	✓	✓	^145,150,426^
Irisin*	Skeletal muscle and adipose	✓	✓	✓	^30,180,183,288,427–429^
ANGPTL4*	Skeletal muscle, adipose, hepatic	✓	✓	✓	^97,243,244^
Adiponectin*	Adipose	×	✓	✓	^430–432^
Apelin*	Adipose	×	✓	✓	^147,152,433,434^
TGF-β2*	Adipose	✓	✓	✓	^151^
RCN2	Bone marrow macrophages	×	✓	×	^124^
METRLN	Macrophages	✓	✓	×	^128,186,435^
IL-10*	Macrophages	✓	✓	✓	^436–439^
IL-1ra*	Blood mononuclear cells	✓	✓	✓	^438–440^
Klotho*	Kidney	✓	✓	✓	^132,133,441^
SDC4*	Hepatic	✓	✓	✓	^135,442^
BDNF*	Nervous, skeletal muscle	✓	✓	✓	^127,198,443,444^
NRG1*	Endothelium	×	✓	×	^176,445^
Decorin*	Skeletal muscle	✓	✓	✓	^337,446^
Cathepsin B*	Skeletal muscle	✓	×	✓	^204,447^
GPLD1*	Hepatic	×	×	✓	^210^
PF4	Platelets	×	×	✓	^205–208^
Adropin*	Hepatic	✓	×	✓	^248,249,448,449^
Clusterin*	Unclear	×	×	✓	^209,450^
HSPs*	Various	✓	✓	✓	^451,452^
Tissue metabolites and lipids					
12,13-diHOME*	Adipose	×	✓	×	^56^
KYNA*	Skeletal muscle	×	✓	✓	^185,453^
NO*	Endothelium	✓	✓	✓	^261,454^
ROS*	Skeletal muscle	✓	✓	✓	^455^
Catecholamines*	Adrenal gland	×	×	✓	^456^
Lactate*	Skeletal muscle	✓	✓	✓	^41,44,457^
Succinate*	Skeletal muscle	✓	✓	✓	^52,55^
L-BAIBA*	Skeletal muscle	✓	✓	✓	^57,58^
Lac-Phe*	Monocytes, macrophages, epithelial cells	×	×	✓	^17^
Nucleic acids					
microRNAs*	Skeletal muscle	✓	✓	✓	^458–460^
lncRNAs*	Skeletal muscle	✓	×	×	^82,461^

The selection of exerkines is based on in vivo evidence of signaling molecules that are mobilized in response to acute exercise or exercise training and signal to target tissues. In vitro studies and studies employing external administration of exerkine isolates were not considered. Exerkines that have been investigated in humans are marked with an asterisk

*ANGPT1* angiopoietin 1, *FGF21* fibroblast growth factor 21, *GDF15* growth differentiation factor 1, *IL-6* interleukin 6, *IL-7* interleukin 7, *IL-8* interleukin 8, *IL-15* interleukin 15, *NTN* neurturin, *SPARC* secreted protein acidic and rich in cysteine, *VEGF* vascular endothelial growth factor, *FST* follistatin, *FSTL1* follistatin-like 1, *ANGPTL4* angiopoietin-like 4, *TGF- β2* transforming growth factor β-2, *METRLN* Meteorin Like, Glial Cell Differentiation Regulator, *RCN2* reticulocalbin 2, *IL-10* interleukin 10, *IL-1ra* interleukin 1 receptor antagonist, *SDC4* syndecan 4, *BDNF* brain-derived neurotrophic factor, *NRG1* neuregulin 1, *HSP* heat shock protein, *GPLD1* glycosylphosphatidylinositol-specific phospholipase D1, *PF4* platelet factor 4, *12,13-diHOME* 12,13-dihydroxy-9Z-octadecenoic acid, *KYNA* kynurenic acid, *NO* nitric oxide, *ROS* reactive oxygen species, *L-BAIBA* β-aminoisobutyric acid, Lac-Phe Lactoylphenylalanine, *lncRNA* long non-coding RNA

#### Secretion via extracellular vesicles

A relatively new area of research in the context of exercise is centered around the release of different exerkines in the form of EVs. EVs are defined as secreted membranous structures containing a cargo and are subdivided into exosomes, microvesicles, and apoptotic blebs based on their size and biochemistry (i.e., expression of specific proteins and lipids).^12,13^ Of note, this classification lacks unambiguity^12,108^ and the different types of EVs most likely depict a continuum rather than strictly separated categories. Methods that enable the characterization of EVs are rapidly evolving given the diagnostic and therapeutic potential of these sub-cellular structures in fields as oncology,^109^ neurology,^110^ and immunology.^111^ Different state-of-the-art technologies and crucial considerations in EV analysis were recently reviewed comprehensively by Hendrix et al.^112^ The special focus on EVs in the context of exercise has arisen from the finding that many exerkines are contained in exosomes, when cross-validating with publicly available databases for EVs such as ExoCarta, Vesiclepedia, or EVpedia.^12,13^ The concentration of EVs in circulation increases after a single bout of acute exercise,^113,114^ thus conferring inter-organ crosstalk between a wide range of tissues^115–118^ with potential implications for tissue adaption to exercise.^12,13,119^

Besides peptides, proteins, tissue metabolites, and lipids, EVs also depict a crucial mode of transport for non-coding RNAs such as miRNAs and lncRNAs.^120–122^ For instance, EV-contained miR-10b-5p, miR-222-3p, and miR-30a-5p were increased transiently after acute exercise and originated from different cell types including endothelial, epithelial, immune, and muscle cells.^123^ An intrinsic property of EVs lies in their membranous structure which protects the contained cargo (e.g., exerkines) from enzymatic degradation in the extracellular space and/or the bloodstream, thus enabling communication between distant tissues despite hostile surrounding conditions. Therefore, EVs depict an important mechanism for exerkine secretion, tissue crosstalk and exercise adaptions. The emerging field of research on exercise-mobilized EVs has crucial implications as molecular framework for tissue-wide health effects of exercise and future research will have to show how the mechanistic insights into exercise-induced EV trafficking can be harnessed therapeutically.

### Distribution of exerkines

Once exerkines are secreted they can signal to the tissue they are released from in an autocrine manner or to distinct target tissues in a paracrine and/or endocrine manner. The exact fate of different exerkines depends on the molecular structure and mode of secretion, as well as the mechanisms governing the interaction with target cells. Systemic distribution via the bloodstream, as displayed by lactate, renders exerkines available to many potential binding sites, thus conferring a plethora of effects in spatially separated tissues.^44,45^ Conversely, tissue crosstalk may also take place in a more localized manner, as exemplified by the paracrine secretion of reticulocalbin-2 (RCN2) from bone marrow macrophages to nearby adipocytes.^124^ For the secretion of exerkines in EVs both systemic (endocrine) and local (auto-/paracrine) mechanisms might apply.

A further important aspect of exerkine distribution is the ability of some exerkines to cross physiological barriers such as the blood-brain barrier (BBB). Knowledge of barrier permeability is crucial, especially when investigating potential health effects of exerkines on the central nervous system (CNS). For instance, many efforts were made to link exercise-induced increases in irisin to central effects such as the release of brain-derived neurotrophic factor (BDNF), synaptic plasticity, and neuronal survival with potential application in neurodegenerative diseases like Alzheimer’s disease (AD) or Parkinson’s disease.^125–127^ However, a crucial caveat of these efforts concerned the question whether irisin mobilized from skeletal muscle tissue was able to confer effects inside the CNS, i.e., whether peripheral irisin was able to cross the BBB. In two landmark studies, Wrann et al. demonstrated that both, central and peripheral increases in irisin mediate cognitive benefits of exercise, suggesting that irisin originating from the periphery is able to enter the CNS.^125,127^ A tabular overview of different exerkines, their source tissues, and their mode of intercellular communication is given in Table 1.

**Fig. 1:**
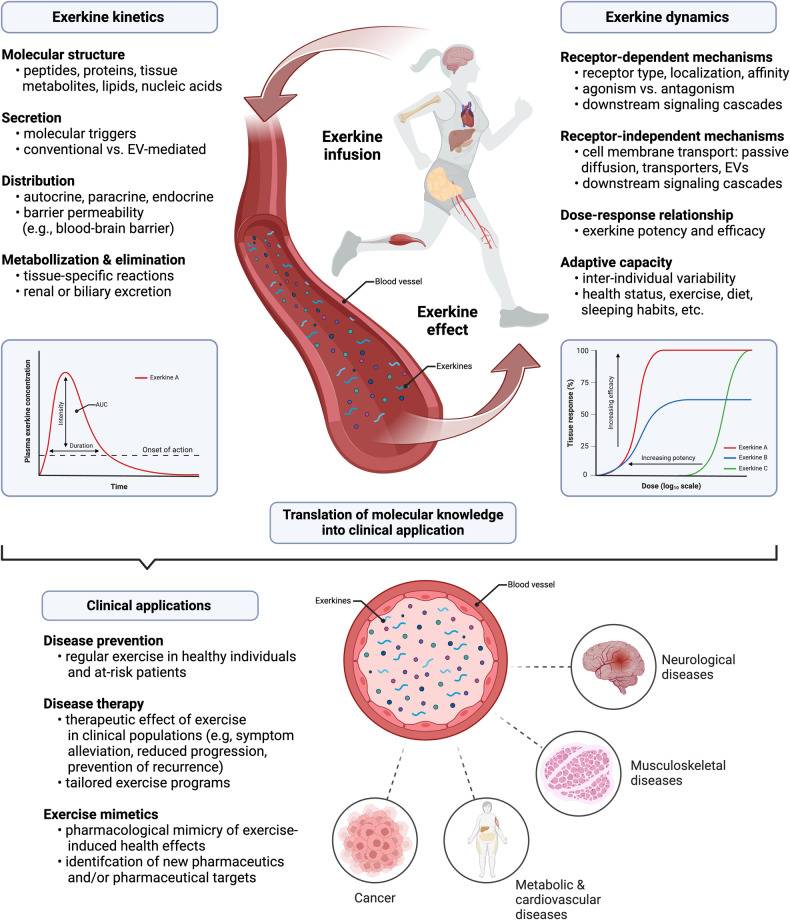
Molecular exercise therapy: mode of action and clinical implications of exercise-induced signaling molecules (exerkines). The effect of exerkines on the human organism can broadly be divided into exerkine kinetics and exerkine dynamics. During acute exercise, numerous exerkines are secreted in an autocrine, paracrine and/or endocrine manner. In the case of endocrine secretion, these exerkines are distributed throughout the human organisms, making them available to distinct target tissues. The intensity and duration of an exerkine effect is dictated by the exerkine concentration over time (area under the curve, AUC), which, in case of endocrine secretion, can be quantified as plasma exerkine levels. For autocrine and paracrine secretion, microdialysis or other techniques for isolation of extracellular fluids^404^ allow precise quantification of tissue-specific exerkine concentrations and determination of exerkine concentration-time curves. Of note, exerkines might also be subject to metabolization and elimination via distinct routes. Once exerkines are secreted, they interact with target cells in a receptor-dependent or receptor-independent manner. For receptor-dependent interactions, the effect on target cells depends on the precise characteristics of the target receptor and the exerkine–receptor interaction. The intrinsic activity of an exerkine (agonism vs. antagonism), its affinity to the target receptor, and the receptor density on target cells dictate dose-response relationships for exerkine-exerkine receptor pairs that determine the potency and efficacy of an exerkine. For receptor-independent mechanisms, passive diffusion across the cell membrane, transmembrane transporters, and extracellular vesicle (EV)-mediated uptake of exerkines have been described. Once exerkines have entered the intracellular space, they can trigger signal transduction and subsequent adaption processes in a distinct fashion. These molecular characteristics as well as inter-individual differences in health status and lifestyle habits (e.g., diet, exercise, sleeping behavior) determine the magnitude of tissue adaption. Transferring mechanistic knowledge on exerkine kinetics and exerkine dynamics into disease context has promising clinical implications, e.g., in disease prevention, targeted exercise therapy and the development of novel, exercise-inspired pharmaceutics (i.e., exercise mimetics). Created with BioRender.com

Collectively, the molecular structure of different exerkines, their mode of secretion, and their local or systemic distribution throughout the organism form the molecular foundation for exercise-mediated health effects across distinct target tissues. The aspects covered in this section of the review can be regarded as the first step in a sequence of molecular events that ultimately result in cellular adaption. As such, *exerkine kinetics*, in analogy to *pharmacokinetics*, can be regarded as the interaction of the human organism with an exerkine—i.e., secretion, distribution, metabolization, and elimination of exerkines. Conversely, *exerkine dynamics*, in analogy to *pharmacodynamics*, describes the interaction of an exerkine with the human organism—i.e., receptor-dependent, and receptor-independent cell signaling, dose-response relationships, and tissue adaptions induced by exerkines. In separating these molecular events and highlighting a clear sequence, we provide a physiological basis for exercise-mediated health effects, which form an indispensable foundation for the prevention and treatment of various chronic diseases (Fig. 1).

## Exerkine-induced signal transduction and biological tissue adaption

After mobilization and distribution of exerkines they can act on target cells in distinct fashion. On the one hand, exerkines can interact with membrane-bound receptors on target cells, thereby triggering cellular signaling cascades that ultimately result in altered gene expression and cellular adaption. On the other hand, exerkines can also interact with target cells in a receptor-independent manner, as exemplified by the delivery of proteins, tissue metabolites, lipids, and non-coding RNAs to target cells as EVs. In contrast to these direct effects of exerkines on target cells, exerkine signals might also be forwarded by one cell type to other cells (e.g., via secretion of cytokines), thus conveying exerkine-mediated adaption processes in an indirect manner. Immune cells are a prime example for these indirect effects since they can move freely between the bloodstream and peripheral tissues and are therefore capable of triggering tissue adaption after stimulation through exerkines. For instance, meteorin-like (METRNL), an exercise-responsive myokine and cold-sensitive adipokine, promotes immune cell infiltration into adipose tissue of mice, and triggers the secretion of interleukin-4 from eosinophils, which contributes to beiging of adipose tissue.^128^ Similarly, RCN2, a mechanosensitive factor released from bone marrow macrophages in response to exercise, induces lipolysis in bone marrow adipocytes, which in turn fuels osteogenesis and lymphopoiesis.^124^ These examples demonstrate that exerkine receptor expression is not a prerequisite for cellular adaption to exercise.

**Fig. 2:**
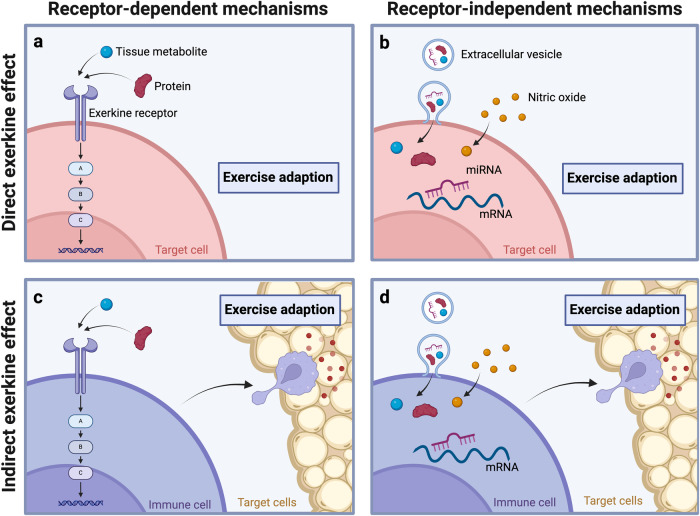
Signaling mechanisms of exerkines. Exerkines may mediate cellular adaption via direct action on target cells (direct exerkine effect) or by stimulating other cell types to release bioactive compounds such as cytokines (indirect exerkine effect). Both, direct and indirect effects require the interaction of exerkines with target cells as an initial step. **a**, **b** In the case of direct exerkine effects, the cellular adaptions occur within the target cells themselves. **c**, **d** For indirect exerkine effects, the targeted cells induce adaption processes in other cell types. Interaction of exerkines with target cells can occur in a receptor-dependent (**a**, **c**) or a receptor-independent manner (**b**, **d**). Extracellular vesicles and nitric oxide are prime examples of receptor-independent exerkine mechanisms. Created with BioRender.com

Whether exerkines act on target cells in a direct manner or stimulate other cell types (e.g., immune cells, endothelial cells) to induce tissue adaption, both mechanisms are dependent on receptor-dependent or receptor-independent signal transduction of exerkine signals as an initial step. Receptor-dependent signal transduction has received overwhelming scientific attention, partly because the identification of molecular exerkine targets (i.e., exerkine receptors) depicts an attractive approach for the design of novel therapeutics. As such, several pharmaceutics have found their way into disease therapy.^25^ However, considering exercise therapy and the associated organism-wide health effects, receptor-independent mechanisms might be of equal relevance. A schematic representation of the different signaling mechanisms harnessed by exerkines is given in Fig. 2.

### Receptor-dependent signal transduction

In terms of accessibility, membrane-bound receptors are the first and most easily reached targets of exerkines. Identifying cellular receptors as molecular transducers of exerkine signals has thus been a major quest of mechanistic exercise research. Many of the identified exerkine receptors belong to well established receptor families like G protein-coupled receptors, tyrosine kinase receptors, or cytokine receptors with tissue-specific expression patterns, respectively (searchable on databases like The Human Protein Atlas or GeneCards).^129^ In contrast, the molecular targets of some exerkines—e.g., 12,13-diHOME,^56^ SPARC,^130,131^ Klotho,^132,133^ Lac-Phe,^17^ Myonectin,^134^ and SDC4^135^—are yet to be discovered. To appreciate the cellular signaling cascades triggered by exerkine receptors as well as translational research in human populations, we have separated in vitro and animal studies investigating exerkine-mediated tissue adaption from human exercise trials.

#### In vitro studies

Frequently used approaches in biomedical exercise research aimed at investigating the effect of exerkines on a target tissue of interest are cell culture experiments with exerkine isolates^136^ or exercise-conditioned serum.^137^ Exercise-mimicking interventions such as electrical pulse stimulation or mechanical stretch of skeletal muscle cells additionally enable investigation of exercise effects in vitro.^138,139^ While such methodological approaches allow for mechanistic conclusions, a major limitation is their poor resemblance of multi-tissue complexity and inter-organ crosstalk, which marks higher living organisms. Although in vitro co-culture models and medium transfer between different cell cultures provide a slight remedy in this regard, animal models depict a pivotal resource to investigate inter-tissue crosstalk triggered by exercise.

#### Animal studies

With increasing scientific interest in the health benefits of exercise, numerous animal models have been introduced, ranging from smaller animals such as *Drosophila melangolaster*^140^ and *Caenorhabditis elegans*^141,142^ to larger animals like mice, rats, dogs, pigs or horses.^143^ Crucial aspects concerning the correct choice of animal model and the appropriate implementation of exercise regimens were recently pointed out to provide methodological guidance in animal research and improve reproducibility.^143^ In terms of tissue-specific effects of exerkines, genetically modified organisms (i.e., knockout, knockdown or transgenic, knock-in) and pharmacological blockade of exerkine receptors depict valuable strategies to investigate exerkine receptor signaling and tissue adaption in vivo. Of note, animal studies are often complemented by in vitro experiments to enable mechanistic conclusions on cellular signaling cascades triggered by exerkines. For instance, the combination of cell culture experiments and animal knockout models identified IL-15 as a muscle-secreted exerkine that attenuates skin ageing.^144^ To summarize the distinct effects of exerkines on different target tissues, we have condensed the current knowledge on receptor-dependent signal transduction and biological tissue adaptions derived from animal studies by tissue type.

##### Skeletal muscle

The prestigious history of myokines as signaling molecules secreted by contracting skeletal muscle has led to numerous studies investigating muscle-specific exerkine receptors.^95,145–154^ These investigations shifted the perception of skeletal muscle from having merely endocrine and/or paracrine function to serving as a direct target of these myokines as well (i.e., autocrine stimulation).

For instance, the neurotrophic myokine neurturin was recently shown to trigger similar signaling cascades in both, adjacent motor neurons and skeletal muscle tissue itself via a target receptor complex between glial cell line derived neurotrophic factor (GDNF) family receptor alpha 2 (GFRA2) and RET receptor tyrosine kinase (GFRA2/RET). Additionally, neurturin can signal via neural cell adhesion molecule (NCAM), with both receptor signaling pathways converging on ERK1/2. Aside from canonical RAF/MEK/ERK signaling, neurturin signaling via GFRA2/RET also led to a transient phosphorylation of AKT and signal transducer and activator of transcription (STAT)3, suggesting the simultaneous involvement of several signaling pathways in skeletal muscle tissue. Ultimately, these signaling cascades coupled the functional identity of motor neurons and skeletal muscle fibers and conveyed typical adaptions to endurance exercise on both, the neuronal, and muscular side of neuromuscular junctions.^146^

Similarly, autocrine stimulation of skeletal muscle tissue was reported for the exerkine apelin via its corresponding apelin receptor (APLNR). After observing that reduced apelin levels were specifically associated with sarcopenia in an elderly population, the protective role of apelin in age-associated skeletal muscle atrophy was deemed a promising research avenue. Apelin secreted from contracting skeletal muscle stimulated muscle APLNR in an autocrine fashion, and led to mitochondrial biogenesis, protein synthesis and satellite cell differentiation in sarcopenic muscle fibers. These anabolic effects were mediated by AMPK, AKT, and the 4E-BP1 cascade, which counteracted the structural decline of muscle tissue observed during inactivity and/or ageing.^152^

Besides these anti-sarcopenic effects, muscle APLNR signaling showed metabolic effects as well.^147^ Increased plasma apelin levels observed in obese and type 2 diabetic subjects led to the hypothesis that apelin secreted from white adipose tissue (WAT) acts as auxiliary glucose-lowering mechanism. Indeed, exogeneous administration of apelin lowered blood glucose levels in normal weight and obese insulin-resistant mice, thereby shedding light at the potential therapeutic implications of apelin in metabolic diseases such as type 2 diabetes mellitus (T2DM). In a series of in vitro and in vivo pharmacological and genetic approaches, this mechanism was shown to be dependent on AMPK-mediated phosphorylation of AKT and endothelial nitric oxide synthase (eNOS), thereby revealing an intersection of canonical insulin signaling via PI3K and apelin signaling at the level of AKT. However, whether AKT is an intermediate between AMPK and eNOS or acts as a separate activator of eNOS remains unclear.^147^ Mechanistically, it was recently shown that exercise-induced AMPK activation in skeletal muscle is dependent on the innate immune sensor toll-like receptor 9 (TLR9) and the core autophagy protein beclin 1. In detail, endogenous ligands like mitochondrial DNA associate with TLR9 during acute exercise and simultaneous interaction of TLR9 and beclin 1 facilitates AMPK activation, plasma membrane GLUT4 localization, and glucose uptake.^155^

The great scientific interest in exercise-induced signaling molecules with glucose-lowering effects^145,147,148,150,151,153,154^ was particularly fostered by the fact that hyperglycemia is associated with an increased risk for various diseases.^156,157^ Understanding insulin-independent mechanisms of glucose uptake by skeletal muscle in response to exercise was therefore considered a topic with high therapeutic potential. For instance, adiponectin, a glycoprotein secreted mainly from WAT, was found to have anti-diabetic effects, and improve insulin sensitivity via its action on adiponectin receptor (ADIPOR) 1 and 2 in skeletal muscle and liver tissue, respectively. As indicated by affinity assays, these effects are preferably mediated by full-length adiponectin in hepatocytes and globular adiponectin, a cleavage product of full-length adiponectin, in myocytes.^153^ In myocytes, ADIPOR1 signaling activates AMPK,^153^ while in hepatocytes ADIPOR1 and ADIPOR2 singling activates AMPK and peroxisome proliferator-activated receptor (PPAR) α signaling pathways.^154^ Additionally, adiponectin increases mitochondrial content of myocytes via calcium influx, calcium/calmodulin-dependent protein kinase kinase beta (CaMKKβ), AMPK, and SIRT1, which contribute to peroxisome proliferator-activated receptor gamma coactivator (PGC) 1-α expression and activation (i.e., phosphorylation, deacetylation).^148^ The fact that both, metabolic and morphologic adaptions of muscle cells are mediated by ADIPOR1 signaling, suggests that similar adaptions also occur in response to exercise; however, such investigations are lacking so far.

Similar anti-diabetic effects were found for transforming growth factor beta-2 (TGF-β2), another exerkine secreted by WAT.^151^ In search of the molecular foundation for increased serum TGF-β2 levels observed after exercise training in humans and mice, muscle-secreted lactate was found to mediate TGF-β2 secretion from subcutaneous WAT in mice. Besides a potential autocrine stimulation of WAT, which leads to adipose tissue browning,^158^ secretion of TGF-β2 from WAT induced glucose and fatty acid uptake in skeletal muscle, improved glucose tolerance and insulin sensitivity and reduced fat inflammation in mice.^151^ Although the cellular mechanisms mediating these effects in the context of exercise remain uncertain, it is tempting to speculate that MAPK and SMAD proteins are involved.^159^ These two examples, adiponectin and TGF-β2, shed light on the endocrine communication between WAT and skeletal muscle tissue and exemplify how inter-organ crosstalk mediates health benefits of exercise via the regulation of systemic metabolism.

Besides the studies highlighted above, further exerkine receptors studied in in the context of skeletal muscle tissue adaption to exercise are IL-15 receptor alpha (IL-15Rα) and natriuretic peptide receptor 3 (NPR3). Despite the multi-faceted functions of IL-15,^160^ and evidence of secretion from skeletal muscle tissue in response to exercise,^144,161,162^ few studies have linked exercise-mediated increases in IL-15 to tissue adaption in vivo. Genetic overexpression of IL-15 in skeletal muscle tissue has shown to confer resistance to diet-induced adiposity and increase bone mineral content of transgenic mice.^163^ In humans, plasma IL-15 levels were negatively associated with fat mass,^164^ however, a mechanistic connection between exercise, IL-15 secretion, and reduction of fat mass is still lacking. In contrast, a clear mechanism of action for muscle-derived IL-15 in morphologic adaptions of skin tissue was revealed by Crane and colleagues. In detail, IL-15 triggered mitochondrial biogenesis in a PPARɣ- and STAT5-dependent manner, thereby attenuating skin ageing.^144^ Surprisingly, ablation of IL-15Rα primed skeletal muscle of mice to a state consistent with moderate exercise, suggesting IL-15 receptor blockade as an exercise mimetic strategy.^149^ These inconsistent results highlight that the distinct functions of IL-15 in skeletal muscle tissue adaption to exercise are yet to be uncovered.

In contrast, musclin, a further muscle-secreted exerkine, mediates skeletal muscle tissue adaption in a distinct manner. Exercise-induced expression of musclin was shown to depend on calcium-dependent phosphorylation of AKT and reduced levels of nuclear forkhead box protein (FOXO) 1, an inhibitor of musclin transcription.^95^ Due to its structural homology to natriuretic peptides (NP) such as atrial natriuretic peptide (ANP), musclin competes for the NP clearance receptor NPR3 (also named NPRC), which does not exhibit cytoplasmic guanyl cyclase activity and was previously described to tailor NP levels to the local needs of distinct organs.^165^ This competition increases the half-life of ANP due to reduced clearance and triggers the cGMP-dependent expression of PGC-1α mediated by NP receptors (i.e., NPR1, NPR2), which exhibit cytoplasmic guanyl cyclase activity. Of note, these effects were only observed when co-incubating myoblasts with musclin and ANP, which is unsurprising, given the low affinity of musclin for NPR1 and NPR2 compared to NP clearance receptor NPR3. Ultimately, ANP/cGMP/PGC-1α signaling led to higher expression of PGC-1α target genes, increased mitochondrial content, and higher exercise performance in wildtype compared to musclin knockout mice.^95^

In summary, skeletal muscle is stimulated by various exerkines in an autocrine, paracrine, and/or endocrine fashion and the structural and functional adaptions induced by exerkine signaling range from classic endurance adaptions,^95,146,148,149,152^ via anti-sarcopenic effects,^51,52,152,166^ through to anti-diabetic and metabolic effects.^145,147,148,150,151,153,154^ Besides mechanistic insights into the molecular foundations of exercise adaptions, these studies highlight how the knowledge of specific exerkine receptor signaling in a target tissue can be transferred to potential other target tissues that express the same exerkine receptor.^146,147,152^ Adopting a hypothesis-driven research strategy and maintaining a clear focus on the origin and potential targets of established exerkines holds the potential to advance our understanding of the pan-tissue benefits of exercise with potential clinical implications in different diseases.

##### Cardiac muscle

Cardiac muscle undergoes considerable amounts of mechanical and metabolic stress during acute exercise, resulting in numerous adaptions including increased mitochondrial density, fatty acid oxidation, and ATP levels as well as improved ROS scavenging and heart contractility.^24^ Sympathetic activation increases heart rate and heart contractility via β2-adrenergic receptors, which leads to an augmented cardiac output, thereby enhancing systemic oxygen and nutrient supply.^167^ Recurring exposure to exercise stress results in myocardial hypertrophy which increases stroke volume and many other aspects of cardiac function, thereby improving exercise performance. Although the global mechanisms of cardiac exercise adaption are mediated in large part by well-investigated growth factors such as insulin, insulin-like growth factor 1 (IGF-1) or VEGF,^168^ various other signaling molecules were shown to contribute to cardiac tissue adaption in response to exercise.

Apelin signaling via cardiac APLNRs seems to be involved in both, the physiological induction of myocardial hypertrophy as well as its amelioration in diseases such as hypertension, T2DM, or obesity. Several signaling cascades have been proposed for these adaptions, including the PI3K/AKT/ERK pathway, mTOR and reactive oxygen species (ROS) signaling, as well as repression of TGF-β1 and hydrogen peroxide (H_2_O_2_) signaling.^169^ This suggests a context-dependent role of APLNR signaling in cardiac exercise adaptions, although to date, exercise models have not confirmed this. The fact that apelin targets skeletal muscle and potentially cardiac muscle via APLNRs, exemplifies how the same exerkine can trigger tissue-specific adaption processes in very different tissues via the same exerkine receptor, thereby underlining the pan-tissue effects that exerkines can exert on the human organism.

Under physiological conditions, exercise-induced myocardial hypertrophy is dependent on parallel lymphangiogenesis via activation of VEGF receptor 3 (VEGFR3).^170^ Pharmacological blockade of VEGFR3 resulted in lower secretion of IGF-1 and reelin, both of which seem to mediate crosstalk between lymphatic endothelial cells and cardiomyocytes. Mechanistically, conditioned medium from lymphatic endothelial cells induced cardiomyocyte hypertrophy via AKT activation and the C/EBPβ-CITED4 axis in vitro. These signaling pathways were also activated after exercise in cardiac tissue of vehicle treated mice compared to mice injected with a VEGFR3 inhibitor.^170^ On the one hand, these results highlight that exercise adaptions are rarely targeted to a specific tissue type (i.e., cardiac muscle), but rather depend on inter-tissue crosstalk via distinct signaling molecules. On the other hand, they reinforce the notion that different exerkines (e.g., apelin and VEGF) harness separate signaling mechanisms to serve a mutual adaption (i.e., myocardial hypertrophy). Of note, the fact that different exerkines transduce their signals via distinct receptors does not rule out the possibility that the triggered signaling cascades might converge at some point to facilitate mutual tissue adaption.

In a more disease-specific setting exercise training improved cardiac function after myocardial infarction in rats.^171^ The increased levels of mature BDNF found in skeletal muscle and non-infarcted areas of the left ventricle suggested that exercise-induced increases in BDNF could exert cardioprotective effects.^172^ Tropomyosin-related kinase B (TRKB) was found to transduce BDNF signals and improve cardiac function through downstream calcium/calmodulin-dependent protein kinase II (CaMKII) and AKT phosphorylation,^171^ thereby emphasizing how exerkine signaling is not only involved in cardiac tissue adaption under physiological conditions, but can also exert regenerative effects in tissue pathophysiology. AKT is also activated in cardiac muscle tissue in response to remote ischemic preconditioning performed during coronary artery bypass surgery,^173^ elucidating a previously unknown mechanisms for cardioprotection that warrants further investigation.^174^

Another prominent exerkine that exerts cardioregenerative effects is METRNL, a muscle-secreted protein that was originally discovered in the context of adipose tissue and regulation of energy homeostasis.^128^ In cardiac tissue, METRNL induced angiogenesis and tissue repair via endothelial KIT receptor tyrosine kinase in a mouse model of myocardial infarction.^175^ Although exercise-induced increases in METRNL expression have been reported in humans and mice,^128^ a thorough investigation of cardiac METRNL-signaling in the context of exercise—as done for BDNF/TRKB signaling—has not yet been performed so far and the exact downstream mechanisms through which this activation leads to cardiac repair are still lacking. Neuregulin 1 (NRG1), a growth factor involved in tissue development also functions as an exerkine in cardiac tissue as observed in a rat model of myocardial infarction, in which chronic moderate exercise induced the endothelial secretion of NRG1, favoring cardiac repair and regeneration by promoting DNA synthesis in adjacent cardiomyocytes via activation of Erb-B2 Receptor Tyrosine Kinase 2 and 4 (ERBB2 and ERBB4) receptor and downstream PI3K/AKT signaling.^176^

In conclusion, various exerkines were shown to induce cardiac tissue adaption via their action on specific exerkine receptors. While some of these receptors are involved in physiological signal transduction and adaption processes initiated by exercise, others have also revealed therapeutic and regenerative effects in cardiac tissue pathology. Elucidating the precise molecular interactions that transduce exercise signals into potential therapeutic effects remains an important topic for future investigations on the cardiac implications of exercise training. In this context, exerkine receptors could prove to be a crucial component of signal transduction, that mediates tissue adaption in health and disease. To harness these mechanisms therapeutically, however, pre-clinical and clinical trials are needed to confirm feasibility, safety, and effectiveness in patient collectives.

##### White adipose tissue

WAT was historically perceived as a rather passive tissue that stores energy for times of nutrient scarcity but is nowadays viewed as a cellularly diverse organ with multi-faceted functions. The different cell types found in adipose tissue include pre-adipocytes, beige adipocytes, fibroblasts, endothelial cells, and various types of immune cells, all of which participate in intercellular communication via distinct mediators.^177^ In the context of exercise, a special interest has emerged concerning the morphological transition of white adipocytes towards a brown phenotype—a process known as adipose tissue browning or beiging.^178^ Since excess adipose tissue constitutes a major risk factor for numerous diseases,^179^ exercise-induced signaling molecules that mediate adipose tissue browning were considered a promising research topic with potential implications for metabolic health.

Irisin, a muscle-secreted exerkine that is proteolytically cleaved from the membrane-bound protein FNDC5, was found to induce adipose tissue browning via cAMP and PPARα, thereby reducing obesity and insulin resistance in exercised mice.^30^ Despite showing that plasma irisin levels also increase in humans performing endurance exercise training,^30,180^ doubts were raised concerning the classification of irisin as an human exerkine.^181,182^ Nonetheless, αV/β5 integrins were identified as molecular transducers of the thermogenic effects of irisin on adipocytes.^183^ These effects were mediated by phosphorylation of several downstream targets of canonical integrin signaling, including focal adhesion kinase (FAK), AKT, CREB, and Zyxin in vitro, and led to increased expression of thermogenic genes such as uncoupling protein 1 (UCP1) and iodothyronine deiodinase 2 (DIO2) in primary inguinal fat cells of mice injected with irisin. A recent characterization of the receptor dynamics between irisin and αV/β5 integrins refined the cellular mechanism of action of irisin by revealing that exercise-secreted extracellular heat shock protein (HSP) 90α activates αV/β5 integrins for binding of irisin at a non-canonical binding site.^184^ Of note, the effects on adipose tissue thermogenic gene programs were replicated in this study and shown to depend on both, irisin and HSP90α. This detailed characterization of exerkine-exerkine receptor dynamics exemplifies how mechanistic insights into exerkine receptor activation advances scientific progress on the tissue-wide adaption processes mediated by exercise. Since numerous tissues express αV integrins, β5 integrins, and HSP90α, irisin-mediated effects on tissue adaption depict a promising area of research. Future studies will have to elucidate to what extent these mechanisms translate to other cell types and human populations.

Similar effects on adipose tissue browning were observed for several other exercise-responsive signaling molecules including METRNL,^128^ and KYNA.^185^ After revealing that METRNL did not act on adipocytes directly, the thermogenic effects were shown to depend on paracrine IL-4 and IL-13 signaling from immune cells secondary to their recruitment to adipose tissue.^128^ Although the molecular mechanisms of METRNL-induced immune cell infiltration (e.g., whether immune cells express a receptor for METRNL) remain unclear, the action of IL-4 and IL-13 on adipose tissue was studied thoroughly. IL-4 and IL-13 are cytokines of a macrophage alternative activation program and acted upon adipocytes via IL-4 receptor alpha chain (IL4Rα) and STAT6, triggering the expression of thermogenic genes in wildtype mice compared to IL4Rα-blocked or STAT6 knockout mice.^128^ Of note, these mechanisms also triggered norepinephrine secretion via increased expression of tyrosine hydroxylase, the rate-limiting step of catecholamine production, thereby linking a further potent stimulator of adipose tissue thermogenesis to the METRNL/IL-4/IL-13 axis. These results shed light on the involvement of the immune system in adipose tissue adaptions and emphasizes the crucial role of migrating immune cells in intercellular communication and healthy tissue adaption. Considering that the health effects of MERTNL are well characterized outside of exercise context,^186–188^ (re)evaluation of these effects in exercise settings could prove valuable to assess the health-promoting effects of exercise as a low-cost lifestyle intervention.

For KYNA, G protein-coupled receptor 35 (GPR35) was identified as a molecular target mediating adipose tissue browning via intracellular calcium release, ERK1/2, CREB phosphorylation and PGC-1α1 stabilization, thereby improving energy homeostasis and inflammation in mice fed a high-fat diet.^185^ Additionally, a crosstalk between GPR35 and beta-adrenergic receptors was identified, in that regulator of G protein signaling 14 (RGS14), a gene enriched in KYNA-treated adipocytes, blocked the inhibitory effect of GPR35 on beta-adrenergic signaling. This unleashed adipocyte beta-adrenergic receptor signaling and enhanced adipose tissue browning in vivo. Further exerkines with similar effects on adipose tissue are β-aminoisobutyric acid (L-BAIBA),^58^ and IL-6.^189^

Beyond adipose tissue browning, fibroblast growth factor (FGF) 21, a peptide hormone secreted in response to acute exercise,^190^ was found to exert further metabolic effects on adipose tissue via a receptor complex between FGF receptor 1 (FGFR1) and co-receptor β-Klotho (KLB). FGF21 is involved in the regulation of energy homeostasis, glucose and lipid metabolism, and insulin sensitivity,^191^ which led to the hypothesis that adipose FGF21 signaling could confer some of the metabolic benefits associated with exercise training. Mice fed a long-term high fat diet exhibited FGF21 resistance together with impaired glucose and insulin tolerance as well as increased fasting insulin, triglyceride, and free fatty acid levels.^192^ Exercise training reversed these effects via restoration of FGF21 sensitivity and PPARɣ-mediated upregulation of FGFR1 and KLB in adipose tissue. These effects were abolished in exercised adipose-specific KLB knockout mice, proving that FGFR1/KLB mediates the metabolic benefits observed after exercise training. Intraperitoneal injections with recombinant mouse FGF21 revealed that FGFR1/KLB signaling triggered downstream ERK1/2 phosphorylation in white and brown adipose tissue.^192^ These results highlight the importance of exerkine dynamics in mediating health-related benefits of exercise. In contrast to other studies, which often focus on exercise-induced increases in specific exerkines and their subsequent action on a certain target tissue (exerkine kinetics), the exercise benefits observed in this investigation were mainly triggered by increased receptor expression and improved sensitivity for FGF21 (exerkine dynamics). Both aspects of exerkine signaling, i.e., exerkine kinetics and exerkine dynamics, are important to improve our mechanistic understanding of exercise-induced health benefits (see Fig. 1).

In conclusion, the effect of exerkine-mediated adipose tissue adaptions is dominated by investigations on adipose tissue browning. The highlighted investigations on cardiometabolic implications of adipose tissue morphology—i.e., WAT as risk factor versus brown adipose tissue as mediator of metabolic health—have advanced our understanding of exercise-induced adipose tissue adaptions conveyed by exerkines. To leverage these mechanistic findings in cardiometabolic diseases such as obesity or T2DM, translational research approaches are warranted to evaluate the therapeutic potential in humans.

##### Bone and cartilage

Bone formation and remodeling depict physiological processes with high therapeutic potential in age- or disease-related bone loss, as frequently observed in osteoporotic patients. The pro-osteogenic impact of exercise is well established nowadays, as evidenced by numerous investigations on the association between exercise and bone mineral density, a main outcome of bone mass and quality.^193^ Several molecular mechanisms have been suggested to transduce the mechanical stimulus imposed on the skeletal system during exercise into tissue adaption. Aside from mechanical deformation and microdamage, endocrine mediators are also involved in the formation of bone tissue in response to exercise via their action on target receptors.^193^

Well-investigated exerkines with osteogenic impact are L-BAIBA and irisin, both of which also target WAT. L-BAIBA—a nonprotein β-amino acid that is physiologically secreted from trained skeletal muscle through catabolism of valine^58^—exerts protective effects on osteocytes via activation of mas-related G protein-coupled receptor type D (MRGPRD).^57^ Mice subjected to hindlimb unloading revealed increased levels of bone and muscle loss, but these effects were attenuated by simultaneous administrations of L-BAIBA. MRGPRD signaling was shown to mediate these effects in osteocytes by protecting mitochondria from ROS-induced damage and regulating mitochondrial gene expression. This protective effect of L-BAIBA was lost in aged mice due to decreased expression of MRGPRD,^57^ highlighting that alterations in exerkine receptor density on target tissues are a crucial determinant of exerkine signaling in vivo (Fig. 1). Although not investigated in the context of exercise, L-BAIBA was shown to induce mitochondrial biogenesis and improve respiratory function of podocytes via MRGPRD and phosphorylation of AMPK in the kidney.^194^ This suggests a role of L-BAIBA in regulating glomerular function with potential applications in different nephropathies.

Similar results with respect to bone remodeling were obtained for irisin signaling via αV/β5 integrins.^183^ Although previous investigations have revealed a positive effect of low-dose weekly injections with irisin on bone formation in mice,^195^ contrary results, i.e., increased bone resorption, were reported after injecting much higher doses on a daily basis.^183^ A dose-dependent regulation of bone resorption and formation might be assumed, given that the cumulative weekly dose of irisin differed by a factor of approximately 60 between these investigations. While irisin-induced bone formation was conveyed by ERK,^195^ bone resorption resulted from integrin signaling via FAK, AKT, CREB, and Zyxin.^183^ The authors argue that irisin could fulfill a dual function similar to parathyroid hormone, which also resorbs bone tissue in chronically elevated concentrations, buts forms new bone tissue when administered intermittently.^183^ In the context of exercise, intermittent irisin pulses might explain the positive effects of irisin on bone formation. Apart from these pharmacodynamic considerations, the obtained results also hold therapeutic value since pharmacological blockade of αV integrins (or irisin) could be harnessed to dampen bone resorption. This therapeutic approach is backed by preclinical experiments showing complete abrogation of osteoporosis in irisin knockout mice^183^ with potential implications for the treatment of osteoporosis and other diseases marked by bone loss. Collectively, L-BAIBA and irisin exemplify how the very same exerkine can induce several health benefits in seemingly unrelated target tissues (i.e., adipose tissue and bone tissue) and how the precise knowledge of exerkine kinetics and exerkine dynamics can facilitate the development of new therapeutic approaches and drug candidates (Fig. 1).

In contrast to endocrine stimulation of bone tissue via L-BAIBA and irisin, mechanical stimulation depicts another trigger of osteogenic adaption. Peng and colleagues demonstrated that mechanical strain sensed by bone marrow macrophages via mechanosensitive ion channels induced the secretion of a lipolytic factor named RCN2.^124^ Subsequent binding to a functional receptor complex on bone marrow adipocytes consisting of neuropilin-2 (NRP2) and integrin β1 (ITGB1) induced lipolysis and lipid mobilization into the surrounding micro-milieu via canonical cAMP/PKA signaling, thereby fueling energy-intensive processes such as osteogenesis and lymphopoiesis inside the bone marrow.^124^ These strongly mechanistic findings shed light at the local interconnection of different cell types (e.g., adipocytes, osteocytes, lymphoid progenitors) and provide new insights into the role of mechanical loading for bone marrow physiology. The results also reveal that exerkines are not necessarily released into the bloodstream before acting on target tissues but might induce functional adaption locally via paracrine signaling. By identifying different components of exerkine signal transduction—i.e., a new mechanosensitive exerkine (RCN2), the associated exerkine receptor complex (NRP2/ITGB1), and the detailed functional consequences for osteogenesis and lymphopoiesis—the authors show how comprehensive investigations on exercise-related adaption processes can create solid scientific progress in biomedical research. This knowledge of newly identified molecular members of strain-induced osteogenesis and lymphopoiesis opens promising therapeutic options that could be leveraged in osteoporotic or immunocompromised patients.

Besides exerkine effects on bone tissue, there are several investigations that have focused on exerkine-mediated adaptions in cartilage tissue as well. Leveraging transcriptome-wide gene expression analyses, Blazek and colleagues investigated the impact of exercise training on gene expression patterns in cartilage tissue of rats. Interestingly, the exercise program yielded over 600 differentially expressed genes in healthy articular cartilage, many of which clustered around the Gene Ontology (GO)-terms immune response, signal transduction and extracellular matrix biosynthesis.^196^ To gain deeper insights into the intracellular processes that were regulated by exercise training, Kyoto Encyclopedia of Genes and Genome (KEGG) pathway analyses were performed and revealed that signal transduction pathways were a major target regulated by exercise training. In detail, most of the differentially expressed genes were annotated to PI3K/AKT, RAS, RAP1, MAPK, and cAMP signaling pathways, suggesting a strong involvement of these signaling cascades in cartilage tissue adaption to exercise. Although this study did not identify exerkines or molecular targets of exerkines as mediators of cartilage tissue adaption, it provides indisputable evidence for cellular signaling cascades that participate in chondral adaption to exercise.^196^

In summary, several studies have investigated the impact of exerkine receptor signaling on skeletal adaptions. The age- and disease-related decline of bone and cartilage tissue and the associated frailty highlight the clinical implications of exerkine receptors that transduce exercise signals into osteogenic and chondrogenic adaptions. By identifying molecular targets for potential therapeutic use in diseases marked by bone loss, these studies have successfully outlined the translational potential of mechanistic research on exercise adaptions for clinical therapy. Since mechanical strain is an inherent characteristic of most exercise modalities, thorough investigation of mechanosensation by bone tissue and bone marrow resident cells (e.g., immune cells) could prove profitable, especially for elderly populations which bear an increased risk for fractures and infections. To what extent these findings translate to humans, however, remains to be elucidated.

##### Nervous system

While peripheral exerkine target tissues including the peripheral nervous system are easily accessible for exerkines via the blood stream, access to the CNS is more limited due to tight regulation by the BBB.^197^ Biochemical properties including hydrophilicity, lipophilicity, and potential transport mechanisms across the BBB are crucial features that need to be considered when evaluating the impact of exerkines on the CNS. To date, several exerkines have been investigated as signaling molecules that affect the CNS in a receptor-dependent or receptor-independent manner. These include BDNF,^198^ irisin,^125,127,199^ lactate,^200,201^ angiopoietin I (ANGPT1),^202^ GDF15^203^, cathepsin B,^204^ platelet factor 4 (PF4),^205–208^ clusterin,^209^ and glycophosphatidylinositol (GPI)-specific phospholipase D1 (GPLD1).^210^

BDNF, a well-described mediator of neuronal cell survival, differentiation, and plasticity,^211^ that is secreted from skeletal muscle and nervous tissue in response to exercise, was shown to mediate neuroplasticity in the CNS via TRKB singling.^198^ Additionally, endurance exercise increased mouse hippocampus gene expression of FNDC5 (the transmembrane precursor of irisin) via mechanisms dependent on PGC-1α and estrogen-related receptor alpha, which also induced hippocampal gene expression of BDNF.^127^ Peripheral delivery of irisin led to similar increases in hippocampal BDNF expression, suggesting that irisin can pass the BBB. In line with more recent reports on the ability of irisin to cross the BBB,^125,199^ these findings have promising implications for the interconnection of regular exercise and brain health since they establish a causal relationship between peripheral increases in irisin levels (as observed after exercise training) and neurological health benefits mediated by BDNF.^127^

Taking these findings into disease context, reduced irisin levels were found in the hippocampus and cerebrospinal fluid (CSF) of AD patients and in hippocampi of experimental AD models.^126^ Knockdown of brain irisin in mice impaired synaptic plasticity and memory function, both of which were rescued by boosting central or peripheral irisin levels. Exercise-secreted irisin mediated similar neuroprotective effects, and improved memory function in trained mice. Mechanistically, irisin triggered a brain cAMP/PKA/CREB signaling pathway to confer these effects.^126^ Beside consolidating the neuroprotective effect of irisin,^125,127,199^ these findings shed light at the potential therapeutic value of exercise in neurological conditions and raise interesting questions regarding further exerkines that participate in muscle-brain crosstalk (for review see ref. ^212^). Of note, although these investigations did not investigate αV/β5 integrins as molecular targets of irisin,^183^ the ubiquitous expression of integrins across numerous tissues, including the brain, suggests integrin signaling as a potential mechanism for irisin-induced adaptions in the CNS. However, given that different signaling pathways of irisin were found in brain^126^ compared to bone or adipose tissue,^183^ future studies will have to show how irisin exerts its effects across distinct tissues.

Since neurological diseases like AD, vascular dementia, and Parkinson’s disease are marked by cognitive deficits, the impact of exercise training on brain angiogenesis depicts a further mechanism with potential therapeutic value in diseases of the CNS.

Similarly, brain vascularization plays an important role in protection from ischemia, as observed in stroke. ANGPT1, a protein with vascular protective effects^213^ secreted from skeletal muscle tissue in the context of exercise,^214^ was revealed to increase angiogenesis and decrease infarct volume in a rat model of ischemic infarction. These effects were triggered by ANGPT1-dependent activation of Tyrosine-protein kinase receptor TEK (TIE-2) and downstream singling via AKT in the ischemic cortex,^202^ and lead to improved functional outcomes in exercised vs. non-exercised mice.

Similar effects on angiogenesis were found for exercise-induced increases in lactate and the lactate hydroxycarboxylic acid receptor 1 (HCAR1), which is expressed and active in brain tissue.^215^ Exercise training increased capillary density in various brain regions of wildtype, but not HCAR1 knockout mice, indicating that HCAR1 in crucially involved in brain vascularization.^201^ These effects were mediated by ERK1/2 and AKT, which induced hippocampal expression of VEGFA, a potent stimulator of angiogenesis, in exercise trained, lactate-injected wildtype mice compared to HCAR1 knockout controls.^201^ Moreover, exercise-induced increases in lactate conveyed neurogenesis in different brain regions via HCAR1/AKT signaling in wildtype mice compared to HCAR1 knockout mice.^200^ These insights into cerebral vascularization and neurogenesis highlight the preventive and therapeutic value of exercise in neurovascular pathologies, which are often characterized by hypoperfusion and microvascular dysfunction.

In contrast to the CNS, peripheral nerves are not confronted with the caveat of BBB permeability concerning exerkine-induced tissue adaption. Thus, peripheral nerves are readily susceptible to exercise adaptions, as demonstrated by the morphological transition of motor neurons towards a slow type I identity triggered by muscle-secreted neurturin.^146^ Other exercise studies have predominantly focused on peripheral nerve regeneration in response to traumatic nerve injury, although the precise molecular mechanisms mediating these effects remain unknown.^216,217^ A frequently discussed concept involves neurotrophic factors such as glial-derived neurotrophic factor, neurotrophin-3, BDNF, or IGF-1^218,219^ as physiological basis for nerve regeneration via activation of neuronal stem cells.

In summary, muscle-brain crosstalk via exerkines remains a hot topic of research that has opened promising therapeutic avenues for the treatment of different neurological diseases.^212^ The identification of exerkine receptors inside the CNS has clarified our mechanistic understanding of the implications of exercise-mediated health benefits and tissue adaption for brain function and warrants for translational research approaches that elucidate the therapeutic relevance of these findings in clinical populations despite methodological obstacles. Concerning peripheral nerves there is far less evidence for exercise-induced tissue adaption, thus calling for mechanistic studies on the molecular underpinnings of neuronal plasticity in the peripheral nervous system.

##### Concluding remarks on animal studies

**Fig. 3:**
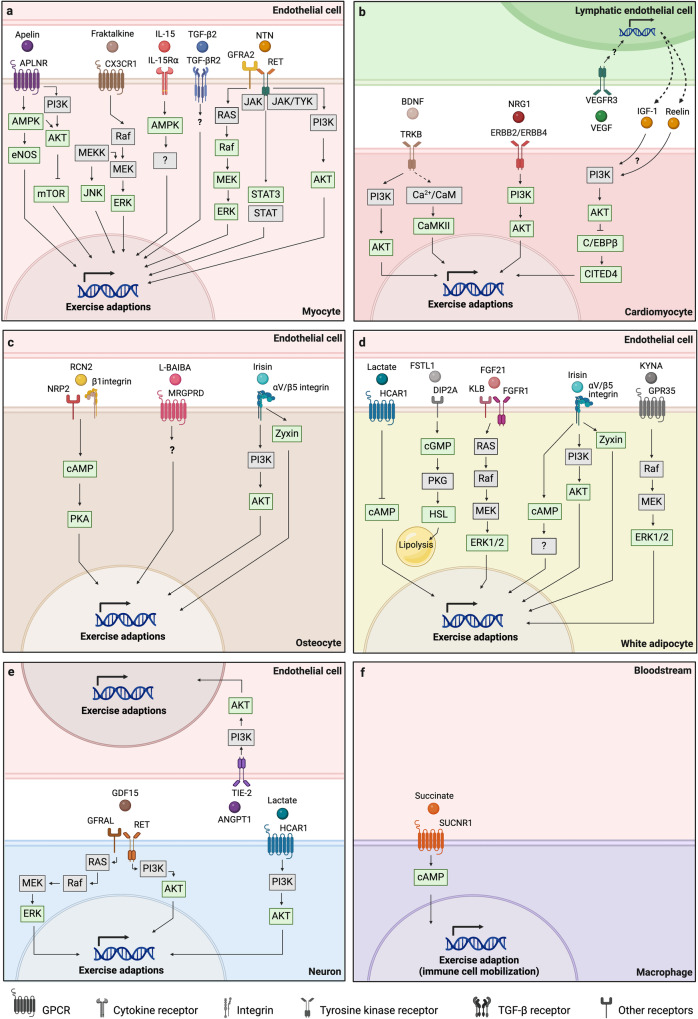
Overview of exerkine receptors and downstream signaling pathways investigated in animal studies. Autocrine, paracrine, and endocrine mobilization makes exerkines available for exerkine receptors localized on distinct target cells including myocytes (**a**), cardiomyocytes (**b**), lymphatic endothelial cells (**b**), osteocytes (**c**), white adipocytes (**d**), neurons (**e**), and macrophages (**f**). Binding of exerkines to their target receptor triggers tissue-specific signaling cascades and adaption processes with potential therapeutic effects in different diseases. Downstream mediators of exerkine signaling that were investigated in vivo are highlighted in green. APLNR apelin receptor, CX3XR1 C-X3-C motif chemokine receptor 1, IL-15Rα Interleukin-15 receptor α, IL-15 Interleukin 15, TGF-βR2 transforming growth factor β receptor 2, GFRA2 Glial cell line derived neurotrophic factor family receptor α 2, RET REarranged during Transfection, NTN Neurturin, TRKB Tropomyosin-related kinase B, BDNF Brain-derived neurotrophic factor, NRG1 Neuregulin 1, ERBB2/ERBB4 Erb-B2 Receptor Tyrosine Kinase 2/ Erb-B2 Receptor Tyrosine Kinase 4, VEGFR3 Vascular endothelial growth factor receptor 3, VEGF Vascular endothelial growth factor, IGF-1 Insulin-like growth factor 1, NRP2 Neuropilin 2, RCN2 Reticulocalbin 2, FSTL1 Follistatin-like 1, DIP2A Disco-interacting protein 2 homolog A, HSL hormone sensitive lipase, MRGPRD Mas-related G protein-coupled receptor type D, L-BAIBA β-aminoisobutyric acid, HCAR1 Hydroxycarboxylic acid receptor 1, FGFR1 Fibroblast growth factor receptor 1, KLB Co-receptor β-Klotho, FGF21 Fibroblast growth factor 21, GPR35 G protein-coupled receptor 35, KYNA Kynurenic acid, GFRAL Glial cell line derived neurotrophic factor family Receptor α Like, GDF15 Growth differentiation factor 15, TIE-2 Tyrosine-protein kinase receptor TEK, ANGPT1 Angiopoietin 1, SUCNR1 Succinate receptor 1. Created with BioRender.com

Exerkine receptor activation was characterized as an important hub between exerkine mobilization and downstream tissue adaption in several animal tissues, including skeletal muscle, cardiac muscle, adipose, bone, cartilage, and nervous tissue. An overview of the target tissues, exerkine receptors, and downstream signaling pathways is given in Fig. 3 and Table 2. Most studies have established a clear connection to exercise, e.g., by training animals with a tissue-specific knockout of a supposed exerkine receptor or by injecting pharmacological exerkine receptor antagonists into exercising mice. These approaches were often accompanied by cell culture experiments to investigate cellular signaling cascades and/or expand the knowledge of established exerkine-exerkine receptor pairs from one tissue to another. Some investigations additionally used human populations as a proof of concept of their findings obtained in rodents. Research approaches like these extend our understanding of the tissue-wide implications of identified exerkines and serve as prime examples of how similar signaling mechanisms might apply to different target tissues. Conceptually, a mutual biological signal—i.e., the mobilization of a certain exerkine—might trigger similar signaling cascades through receptor activation in very distinct tissues. On a broader scale these pan-tissue effects might depict a molecular intersection for the tissue-wide health benefits associated with exercise. The mechanistic insights obtained in animal studies play a crucial role in identifying molecular mechanisms that mediate tissue adaption, however, the successful translation of these findings into human populations is still emerging.

**Table 2:** Examples of receptor-dependent signal transduction and biological effects on target tissues mediated by exerkines

Exerkine	Exerkine receptor	Mechanism of action	Biological effects	Refs
Skeletal muscle				
NTN	GFRA2/RET	RAF/MEK/ERK, AKT, STAT3	↑ vascularization, oxidative capacity, fatty acid transport, mitochondrial metabolism, slow-twitch muscle fibers	^146^
TGF-β2	TGF-βR2	n/a	↑ glucose & fatty acid uptake ↑ glucose tolerance & insulin sensitivity ↓ fat inflammation	^151^
IL-15	IL-15Rα	AMPK	↑ fatty acid oxidation, nucleotide metabolism & anaplerotic reactions	^149^
Fractalkine	CX3CR1	ERK, JNK	↑ neutrophil recruitment, GLUT4 translocation, glucose uptake & myokine secretion	^145,150^
Musclin	NPRC (clearance receptor)	ANP/NPRA or NPRB/cGMP	↑ mitochondrial biogenesis	^95^
Apelin	APLNR	AMPK, eNOS, AKT/mTOR	↑ glucose uptake & glucose tolerance	^147^
			↑ mitochondrial biogenesis, protein synthesis, satellite cell differentiation & protection from sarcopenia	^152^
Cardiac muscle				
BDNF	TRKB	CaMKII, AKT	↑ cardiac function & regeneration after myocardial infarction	^171^
IL-6	IL6R	n/a	↑ exercise-induced cardiac hypertrophy	^225^*
VEGF	VEGFR3	AKT, C/EBPβ-CITED4	↑ lymphangiogenesis contributing to cardiac hypertrophy	^170^
NRG1	ERBB2, ERBB4	PI3K/AKT	↑ cardiac tissue repair & regeneration	^176^
White adipose tissue				
KYNA	GPR35	ERK, CREB, enhanced β-AR signaling	↑ glucose tolerance, lipid metabolism, thermogenic & anti-inflammatory gene expression ↓ weight gain	^185^
FGF21	FGFR1/KLB	ERK1/2	↓ fasting insulin, triglyceride & free fatty acid levels ↑ glucose & insulin tolerance	^192^
Irisin	αV/β5 integrins	cAMP, FAK/AKT/CREB, Zyxin	↑ expression of thermogenic genes & adipose tissue browning	^30,183^
Lactate	HCAR1	cAMP	↓ lipolysis	^462,463^
IL-6	IL6R	STAT3	↓ epi- & pericardial adipose tissue mass	^225^*
		AMPK, STAT3	↑ glucose uptake, glucose & fatty acid metabolism	^295^*
		n/a	↑ thermogenesis	^189^
		n/a	↑ free fatty acid mobilization	^223^*
		n/a	↓ visceral fat mass	^222^*
FSTL1	DIP2A	cGMP	↑ activation of HSL	^32^*
Bone				
Irisin	αV/β5 integrins	FAK/AKT/CREB, Zyxin	↑ osteoclastic bone resorption	^183^
L-BAIBA	MRGPRD	N/A	↑ survival of osteocytes & protection from ROS	^57^
RCN2	NRP2/1 integrins	cAMP/PKA	↑ lipolysis & fatty acid mobilization from bone marrow adipocytes, fueling osteogenesis & lymphopoiesis	^124^
Central nervous system				
Lactate	HCAR1	AKT	↑ neurogenesis	^200^
		ERK1/2, AKT	↑ hippocampal VEGFA & angiogenesis	^201^
ANGPT1	TIE-2	AKT	↑ angiogenesis, cerebral blood flow & functional outcomes ↓ infarct volume	^202^
GDF15	GFRAL/RET	ERK, AKT	↓ food intake & body weight in obese mice	^203^
Immune Cells				
IL-8	IL8R	n/a	↑ prolonged exercise-induced neutrophilia	^232^*
IL-6	IL6R	n/a	↑ exercise-induced mobilization of NK cells & dendritic cells	^224^*
Succinate	SUCNR1	Ca^2+^, cAMP	↑ protein synthesis in skeletal muscle tissue	^51,166^
		n/a	↑ skeletal muscle adaption to exercise	^52^
Skin				
IL-15	IL-15Rα	PPARɣ, STAT5	↑ mitochondrial biogenesis	^144^
Liver				
FN1	α5/β1 integrins	IKKα/β-JNK1-BECN1	↑ hepatic autophagy and systemic insulin sensitization	^250^

The selection of studies displayed in this table is based on in vivo evidence of exerkines with receptor-dependent signaling in response to acute exercise or exercise training. Non-exercise studies were not considered. Human studies involving pharmacological exerkine receptor blockade are marked with an asterisk

*n/a* not available, *APLNR* apelin receptor, *CX3XR1* C-X3-C motif chemokine receptor 1, *IL-15Rα* interleukin-15 receptor α, *IL-15* interleukin 15, *TGF-βR2* transforming growth factor β receptor 2, *GFRA2* glial cell line derived neurotrophic factor family receptor α 2, *RET* REarranged during Transfection, *NTN* neurturin, *TRKB* tropomyosin-related kinase B, *BDNF* brain-derived neurotrophic factor, *NRG1* neuregulin 1, *FN1* fibronectin 1, *FSTL1* follistatin-like 1, *DIP2A* Disco-interacting protein 2 homolog A, *HSL* hormone sensitive lipase, *IL-6* interleukin 6, *IL-R* interleukin 6 receptor, *IL-8* interleukin 8, *IL-8R* interleukin 8 receptor, *ERBB2/ERBB4* Erb-B2 receptor tyrosine kinase 2/Erb-B2 receptor tyrosine kinase 4, *VEGFR3* vascular endothelial growth factor receptor 3, *VEGF* vascular endothelial growth factor, *IGF-1* insulin-like growth factor 1, *NRP2* neuropilin 2, *RCN2* reticulocalbin 2, *MRGPRD* Mas-related G protein-coupled receptor type D, *L-BAIBA* β-aminoisobutyric acid, *HCAR1* hydroxycarboxylic acid receptor 1, *FGFR1* fibroblast growth factor receptor 1, *KLB* Co-receptor β-Klotho, *FGF21* fibroblast growth factor 21, *GPR35* G protein-coupled receptor 35, *KYNA* kynurenic acid, *GFRAL* glial cell line derived neurotrophic factor family Receptor α Like, *GDF15* growth differentiation factor 15, *TIE-2* tyrosine-protein kinase receptor TEK, *ANGPT1* angiopoietin 1, *SUCNR1* succinate receptor 1, *VEGFA* vascular endothelial growth factor A, *NK* natural killer, *ROS* reactive oxygen species

#### Human studies

The direct translation of knowledge derived from animal studies into humans can be difficult, especially in view of methodological and ethical constraints in obtaining certain tissue types in healthy populations (e.g., brain, heart, liver, lung). Other tissues such as subcutaneous adipose tissue, skeletal muscle, or immune cells are better accessible in humans. In clinical settings, incorporation of research projects into surgical procedures might enable access to further target tissues (e.g., tissue biopsies of visceral fat and internal organs), thereby facilitating investigations on the impact of tissue-specific exerkine signaling in the context of different diseases. Despite these implications for human health, only few investigations have made use of such translational approaches. One strategy that has been applied in healthy individuals, however, is the use of exerkine receptor antagonists to investigate the effect of exerkine receptor signaling in humans. The potential of exerkine agonists in conferring healthy tissue adaption (i.e., exercise mimetics) will be covered later in this review. To date, pharmacological inhibition of IL-6 receptor (IL-6R) and IL-8 receptor (IL-8R) has been performed in humans subjected to exercise.

##### IL-6 receptor blockade

Tocilizumab is a humanized monoclonal antibody directed against membrane-bound and soluble IL-6Rs and is therapeutically indicated in several forms of arthritis, giant cell arteritis, and COVID-19 pneumonia.^220^ In human research, tocilizumab can be used to block endogenous IL-6R signaling to investigate the consequences of inhibited signal transduction in vivo. Given that IL-6 is a well-described exerkine with distinct effects on several target tissues,^221^ a randomized, double-blind, placebo-controlled trial performed with abdominally obese participants investigated the impact of IL-6R blockade on adipose tissue in humans.^222^ 12 weeks of progressive endurance exercise significantly reduced visceral fat mass, which was quantified by magnetic resonance imaging (MRI) scans, in exercising participants compared with non-exercising controls. These effects were completely abolished in participants that additionally received tocilizumab.^222^ In fact, while exercising participants receiving placebo infusions achieved an 8% reduction in visceral fat mass, those infused with tocilizumab revealed an increase by 10% despite exercising. Although this does not mechanistically prove that IL-6R signaling is responsible for reductions in visceral fat mass, it highlights a crucial role of this signaling mechanism for visceral fat reduction in abdominally obese humans. Similar results were found in a non-randomized, placebo-controlled crossover trial.^223^ IL-6 receptor blockade with tocilizumab lowered the mobilization of free fatty acids in lean and obese men at rest, during exercise and in the subsequent recovery period, and slightly impaired lipolysis in obese men,^223^ indicating that IL-6R signaling is crucial for adipose tissue homeostasis and systemic energy metabolism both, under resting conditions and in the context of acute exercise.

Based on these studies several secondary analyses were performed to assess the impact of IL-6R signaling on other target tissues and clinical outcomes.^224–226^ Focusing on cardiac implications of IL-6R signaling, a reduction in epicardial fat mass (quantified by MRI scans) was observed in exercising participants receiving placebo infusions compared to non-exercising controls.^225^ These effects were abolished in exercising participants receiving tocilizumab instead. In line with the decrease in epicardial fat mass, left ventricular mass significantly increased in exercising participants receiving placebo infusions, which was abolished in participants receiving tocilizumab. These findings shed light at cardiac exercise adaptions mediated by IL-6R activation and subsequent signal transduction and highlight the scientific potential of imaging techniques such as MRI scans in quantifying adaptions in tissue mass in humans. In another secondary analysis the impact of IL-6R blockade on exercise-induced immune cell mobilization was investigated by analyzing blood samples from an acute exercise bout performed before and after 12 weeks of placebo or tocilizumab infusion.^224^ Peak numbers of circulating natural killer (NK) cells and dendritic cells were decreased in response to acute exercise under IL-6R blockade, suggesting that IL-6R signaling is involved in the exercise-induced mobilization of NK cells and dendritic cells. Animal studies have revealed that exercise-induced IL-6 secretion plays a crucial role in the mobilization and redistribution of NK cells towards tumor tissue, thereby decreasing tumor growth.^227^ However, these effects did not translate to humans, as indicated by two randomized controlled trials with prostate cancer patients.^228,229^ Future studies will have to clarify if these mechanism translate to other tumor entities to elucidate the therapeutic significance of IL-6R signaling in exercise-induced immune cell trafficking.

##### IL-8 receptor blockade

IL-8 was originally discovered as a chemotactic signaling molecule facilitating leukocyte recruitment to sites of inflammation but has also revealed crucial involvement in exercise-induced angiogenesis of skeletal muscle.^230^ These functions are mediated by the IL-8R, which is encoded by the gene C-X-C motif chemokine receptor 2. IL-8Rs are expressed on endothelial cells, and have shown promising results as pharmacological target in various diseases.^231^ To investigate the impact of IL-8R signaling on human neutrophil mobilization, a double-blind, placebo-controlled crossover trial applying pharmacological blockade of IL-8R was conducted.^232^ Circulating neutrophil levels were lower under IL-8R blockade at rest compared to placebo but increased to a similar extent (~40%) in response to acute exercise. The sustained neutrophilia frequently observed after acute exercise^233–235^ was only present in the placebo group, while neutrophil levels returned to baseline under IL-8R blockade. This indicates that IL-8R signaling might be involved in long-term mobilization of neutrophils from the bone marrow, which is suggested as main mechanism for the sustained neutrophilia observed after exercise.^236^ Of note, the statistical significance of these findings remains questionable since no results of statistical analyses were reported. The limited evidence on IL-8R signaling in humans calls for thorough reevaluation of the precise mechanisms intertwining IL-8 secretion and exercise-induced immune cell mobilization.

##### Concluding remarks on human studies

**Fig. 4:**
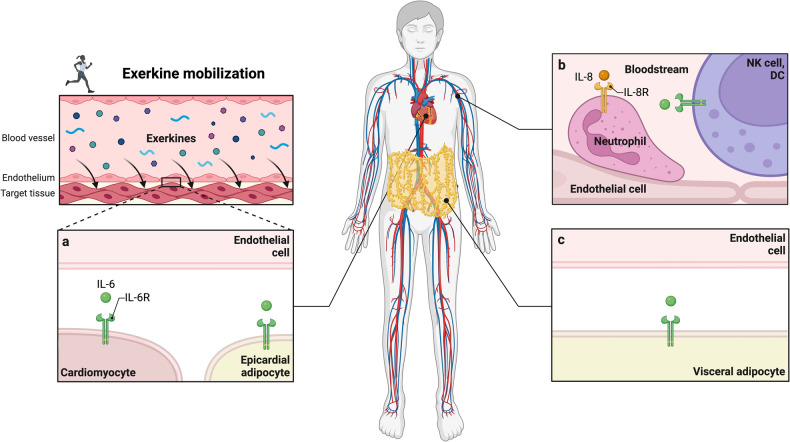
Overview of exerkine receptors investigated in human trials. Due to methodological and ethical constraints of mechanistic exercise studies in humans, exerkine receptor activation is harder to investigate in humans compared to animals. Exerkine target tissues that have been investigated in humans comprise cardiac muscle tissue and epicardial adipose tissue (**a**), neutrophils, natural killer cells, and dendritic cells (**b**), and visceral adipose tissue (**c**). IL-6R interleukin-6 receptor, IL-8R interleukin-8 receptor, NK cell natural killer cell, DC dendritic cell, IL-6R interleukin 6 receptor, IL-8R interleukin 8 receptor. Created with BioRender.com

Despite promising therapeutic implications of exerkine receptor signaling and tissue adaption in vivo, remarkably few studies have translated the molecular mechanisms discovered in animal models into human populations (Fig. 4 and Table 2). The potential of pharmacological exerkine receptor blockade in evaluating the physiological consequences of exerkine signaling in humans was successfully demonstrated by several investigations. Despite ethical hurdles, there are numerous further monoclonal antibodies such as cetuximab (receptor tyrosine kinase antagonist) or fresolimumab (TNF-β receptor antagonist) with potential use in exercise context. An overview of potential blocking agents for membrane-bound receptors was recently provided by O’Shea and colleagues.^237^ To quantify changes in tissue mass in response to exerkine receptor blockade, imaging techniques depict an attractive method in humans. Combining MRI scans,^225^ computed tomography (CT) (as done with micro-CT imaging in animal studies)^124,183^ or positron emission tomography (PET)-CT scans^238^ with pharmacological exerkine receptor antagonists could depict a valuable approach to assess the impact of exerkine receptor signaling on clinical outcomes in humans. Apart from imaging approaches, exerkine receptor activation and the subsequent signal transduction could also be captured via tissue biopsies. In this context, subcutaneous adipose tissue and skeletal muscle are relatively easy to obtain for context-dependent in-depth analyses. Immune cells additionally offer the possibility of ex vivo investigation, e.g., to evaluate the functional consequences of different exerkines and/or exerkine receptor antagonists on cellular signaling cascades. In view of this diverse methodological landscape, translational research approaches applying these methods are needed to create reliable evidence for the health-related impact of exerkines on exerkine receptors and cell signaling in different human target tissues.

### Receptor-independent signal transduction

In contrast to receptor-dependent signal transduction, receptor-independent mechanisms of exerkines are less well investigated due to the more complex nature that governs their action on target cells. Although knowledge on these mechanisms is sparse, we want to point out some prominent examples that exemplify the potential of receptor-independent signal transduction in healthy tissue adaption. This is especially of relevance since these mechanisms might also contribute to the tissue-wide health effects of exercise therapy in different disease settings.

For some of the exerkines discussed previously evidence for membrane-bound receptors as molecular transducers of exercise effects is not available yet. This opens two possibilities: either the receptor exists but has not been linked to the exerkine, or these exerkines act on non-receptor targets directly. For instance, METRNL was shown to promote heart repair and angiogenesis after myocardial infarction via endothelial KIT receptor tyrosine kinase,^175^ but whether the same receptor is responsible for immune cell infiltration into adipose tissue and subsequent cytokine secretion for adipose tissue browning remains unclear.^128^ Other exerkines such as Cathepsin B correlate with memory functions but the precise molecular mechanism are not known yet.^204^

In contrast, follistatin, a liver-secreted exerkine that is regulated by glucagon and insulin levels,^239^ signals to non-receptor targets directly by binding extracellular ligands of activin type II receptors (ACTRII), including myostatin. These ligands usually suppress skeletal muscle anabolism via TGF-β signaling. Conversely, follistatin antagonizes these effects via sequestration of myostatin and other activins, thereby facilitating skeletal muscle hypertrophy^240^ and improving insulin signaling in normal weight and obese mice.^241^ Follistatin thus depicts a prime example of an indirect, receptor-independent mechanism of exerkine signaling (Fig. 2). Besides its action on myostatin and other ligands of ACTRII, follistatin also mediates hypertrophy in a myostatin-independent manner via mechanisms involving SMAD3, AKT, mTOR, S6K, and S6RP, although the precise cellular signaling cascades remain unclear.^242^ Similarly, ANGPTL4, an exerkine secreted by the liver, adipose tissue, and skeletal muscle tissue,^97,243,244^ was shown to activate cAMP/PKA-dependent WAT lipolysis in mice,^245^ but the cellular receptors mediating these effects in adipocytes are unknown. In search for a molecular explanation, extracellular ANGPTL4 was found to potentiate β-adrenergic receptor signaling downstream of the β-adrenergic receptor, but upstream of adenylate cyclase.^245^ However, investigations in the context of exercise have not been performed so far. A further function of ANGPTL4 is the inhibition of lipoprotein lipase in non-exercising muscle tissue, which reduces fatty acid uptake and regulates lipid metabolism. In contrast, exercising muscle tissue is primed for fatty acid uptake, most likely via an AMPK-dependent inhibition of ANGPTL4.^243^ Adropin, a further hepatokine that increases with exercise training, was additionally shown to improve vascular endothelial function in obese adolescents independent of body weight changes.^246^ These effects are most likely mediated by a higher production and bioavailability of NO, resulting in improved endothelial function,^247^ and pro-angiogenic effects in the brain involving VEGFR2 expression and AKT phosphorylation.^248^ An additional role of adropin in nonalcoholic steatohepatitis is suspected due to its suppression of NLRP3 inflammasome components such as caspase-1 and IL-1β.^249^

In contrast to these exerkines, which are secreted from the liver and exert their function in different target tissues, exactly the opposite is true for the fibronectin (FN) 1-α5β1 integrin pathway. FN1 is a muscle-secreted exerkine that binds to α5β1 integrin receptors on the liver, thereby activating hepatic autophagy and systemic insulin sensitization via downstream IKKα/β-JNK1-BECN1 signaling.^250^ These results reveal that non-contractile tissues like the liver are central to metabolic adaptions induced by exercise and that more research is needed on the communicative interconnection of these tissues with contractile tissues like skeletal muscle.

As a metabolic exerkine with high exercise-sensitivity, lactate exerts several effects on target cells in a receptor-independent manner as well. For instance, in mice both, acute exercise, and central lactate infusions resulted in reduced food intake and altered levels of hypothalamic neuropeptide levels, which physiologically regulate appetite via the action of leptin.^251^ Interestingly, these effects were accompanied by increased phosphorylation of hypothalamic JAK2 and STAT3, suggesting a leptin-induced attenuation of appetite via JAK2/STAT3 signaling. By applying intracerebroventricular injections with an LDHA inhibitor, the authors reveal that lactate, a crucial energy source for neurons, sensitizes the action of leptin on neurons for regulation of appetite in the context of exercise.^251^ Apart of these central exercise effects, lactate can also act on peripheral tissues in a receptor-independent manner. For instance, lactate was found to increase adipose tissue browning in a receptor-independent manner via modification of cellular redox balance.^252^ Further examples of receptor-independent actions of lactate comprise the regulation of gluconeogenesis and lactylation of histone residues (for review see ref. ^44^).

HSPs form another class of exercise-responsive proteins that signal to target cells in a receptor-independent manner. Instead of binding to membrane-bound receptors, HSPs—which often originate from within stressed cells themselves—trigger tissue adaption via protein-protein interaction with key players of canonical signal transduction. As such, HSPs are involved in numerous adaption processes across multiple tissues, ranging from skeletal and cardiac muscle, via the lung through to the liver and kidney.^253^ For instance, HSP70 and heat shock transcription factor 1 (HSF1) may explain the protective effect of exercise on pathological cardiac hypertrophy, as observed in hypertension, aortic stenosis, or valvular defects.^254^ Mechanistically, HSF1 leads to reduced expression of NF-κB p65, a key regulator of cardiac hypertrophy and maladaptive remodeling, suggesting that HSP-mediated suppression of NF-κB signaling depicts a molecular mechanisms through which exercise protects cardiac tissue from pathologic adaption.^254^ As for this example, several other HSPs including HSP72^253,255,256^ and HSP27^257,258^ have been linked to functional tissue adaption or health-related benefits in the context of exercise (Table 3).

**Table 3:** Examples of receptor-independent signal transduction and biological effects on target tissues mediated by exerkines

Exerkine	Non-receptor target	Mechanism of action	Biological effects	Refs
HSP72	JNK protein	Protein- protein interaction (JNK inhibition) and/or inhibition of upstream JNK signaling	↑ insulin sensitivity and glucose tolerance	^464,465^
HSP70	NF-κB pathway	Reduced expression of NF-κB p65, inhibition of NF-κB signaling	↓ pathological cardiac hypertrophy	^254^
HSP27	NF-κB pathway	Proteasomal degradation of phosphorylated I-κBα	↓ apoptosis	^257,466,467^
NO	NO-activated guanylate cyclase	Phospho-protein activation via NO/cGMP/PKG pathway	↑ endothelial function, cardiac protection, neuronal plasticity & expression of proteins involved in angiogenesis	^262,468,469^
ROS	MAPK pathway, NF-κB pathway	Phosphorylation/dephosphorylation of key players of signaling pathways	↑ skeletal muscle adaption to exercise	^266–268^
ANGPTL4	Lipoprotein lipase	Inhibition of lipoprotein lipase	Regulation of lipid metabolism in non-exercising vs. exercising muscle	^243^
	β-adrenergic receptors	Potentiates β-adrenergic signaling via cAMP/PKA	↑ WAT lipolysis	^245^
FST	Activin A, myostatin	Sequestration of activin A and myostatin	↑ skeletal muscle hypertrophy & insulin signaling	^241,242^
Decorin	Myostatin	Sequestration of myostatin	↑ skeletal muscle hypertrophy	^337^
miRNAs	Regulation of gene expression	RNA silencing and post-transcriptional regulation of gene expression	Exercise-induced adaptations	^77^
Lactate	Hypothalamic neurons	Serves as energy source for leptin-induced phosphorylation of JAK2/STAT3 in hypothalamic neurons	↓ food intake & exercise-induced suppression of appetite	^251^
	Cellular redox state	Modification of cellular redox state, expression of thermogenic genes	↑ adipose tissue browning	^252^
GLPD1	Membrane-bound protein release	Hydrolyzation of GPI-anchored proteins	↑ hippocampal neurogenesis and cognitive performance	^210^
Adropin	NLRP3 inflammasome	Suppression of NLRP3 inflammasome activation	↓ inflammation in NASH	^249^

All studies listed in this table are animal or in vitro studies

*HSP* heat shock protein, *JNK* c-Jun N-terminal kinases, *NO* nitric oxide, *ROS* reactive oxygen species, *ANGPTL4* angiopoietin-like 4, *FST* Follistatin, *WAT* white adipose tissue, *miRNA* microRNA, *GPI* glycophosphatidylinositol, *GPLD1* glycophosphatidylinositol-specific phospholipase D1, *NLRP3* NOD-, LRR- and pyrin domain-containing protein 3, *NASH* nonalcoholic steatohepatitis

Using a broader definition of exercise-mobilized signaling molecules, nitric oxide (NO) and ROS might also be perceived as exerkines with a receptor-independent mechanisms of action on target cells. Plasma levels of nitric oxide (NO) were shown to increase in response to acute exercise, leading to vasodilation via canonical endothelial NO synthase (eNOS)/cGMP signaling in smooth muscle cells.^259,260^ Exercise training has additionally shown to increase eNOS expression and phosphorylation thereby improving endothelial function, especially in diseases marked by impaired vascular function such as coronary artery disease, atherosclerosis or diabetes mellitus.^261–263^ Given the ubiquitous expression of eNOS and other NOS isoforms (i.e., iNOS, nNOS) across various tissues,^264^ NO-mediated effects of acute exercise and adaptions in response to exercise training have been reported in skeletal muscle tissue as well.^265^ However, the physiological effects of NO extend far beyond what has been investigated in the context of exercise,^264^ thereby creating an urgent need for reevaluation of NO-mediated tissue adaption and the associated health benefits triggered by exercise training. In contrast, ROS is predominantly produced by skeletal muscle tissue during exercise and evokes skeletal muscle adaptions via regulation of distinct signaling pathways, including the mitogen-activated protein kinase (MAPK) family, NF-κB signaling, and PGC-1α.^266^ The precise mechanisms of ROS-induced skeletal muscle tissue adaption are beyond the scope of this review and can be found elsewhere.^266–268^

Regarding indirect effects of exerkines (Fig. 2), exercise-activated platelets are also worth noting since they seem to be involved in the secretion of selenoprotein P (SEPP1), a protein that facilitates transport of the antioxidant selenium to the brain.^269^ SEPP1 binds to the low-density lipoprotein receptor-related protein 8 (LRP8), located on brain capillary endothelial cells, and increases bioavailability of selenium in neural progenitor cells through a so far undescribed mechanism. Once selenium has entered the CNS, it promotes exercise-mediated hippocampal neurogenesis and reduces intracellular ROS in a NOX-2-dependant fashion.^269^ Platelet activation also results in secretion of PF4, which induces transcriptomic changes associated with learning, long-term memory, and synaptic transmission in adult neural precursor cells, thus promoting hippocampal neurogenesis in aged mice. However, the precise signaling pathways mediating these adaptions are not determined yet.^207^

In conclusion, any type of exerkine, including those discussed above, can also signal to target cells via EVs in a receptor-independent manner. Considering the challenging surrounding conditions of the extracellular space and/or blood stream (e.g., changing pH, presence of degrading enzymes, etc.), secretion into EVs might be the only available mode of transport to distant target cells for some exerkines, especially for labile molecules such as RNA.^12^ Once a target cell is reached, EV uptake is accomplished via several mechanisms.^270^ For systemically deployed EVs, paracellular or transcellular transport across the endothelial barrier imposes an additional burden.^271^ A tabular overview of different receptor-independent exerkines together with their mechanism of action, and the associated biological tissue adaption is given in Table 3.

## Exercise therapy in disease prevention and treatment

Regular exercise and physical activity are nowadays viewed as established lifestyle factors with health-promoting effects in disease prevention and treatment. The effects of exercise and physical activity unfold in primary prevention (i.e., protection against the development of disease via modification of risk factors) and tertiary prevention (i.e., the improvement of disease course and clinical outcomes in patients). In contrast to some pharmacological therapies, the mediated effects are not restricted to a specific target tissue, but instead provide clinical benefits in multiple organ systems simultaneously. Physical activity reduces the incidence and mortality of various diseases. For example, greater physical activity levels reduce the risk of coronary heart disease, stroke, diabetes mellitus, colon cancer, and breast cancer,^3^ all of which rank among the top 20 leading causes of death globally.^272^ Similarly, physical activity is associated with a decrease in all-cause, cardiovascular, and cancer mortality.^273,274^ In patients at risk of developing a disease, physical activity has the potential to ameliorate risk factors (i.e., primary prevention), as exemplified by reduced HbA1c and fasting glucose levels, and enhanced oral glucose tolerance in individuals with prediabetes.^275,276^ Once diagnosed, physical activity also exerts tertiary preventive effects, as demonstrated by reduced mortality of patients with coronary heart disease,^277^ stroke,^278^ and chronic obstructive pulmonary disease^279^—the three leading causes of death globally.^272^

Given the large-scale benefits of regular physical activity in promoting health and preventing disease, the World Health Organization (WHO) regularly publishes and updates physical activity recommendations to promote an active lifestyle among the population.^280,281^ In detail, the WHO recommends a minimum of 150–300 min of moderate-intensity aerobic exercise or 75–150 min of vigorous-intensity aerobic exercise per week (or a combination of both) and additional strengthening exercises involving all major muscle groups twice per week. For children, older adults, adults living with chronic medical conditions, and pregnant or postpartum women, these recommendations are expanded and specified accordingly.^280^

### Preventive and therapeutic effects of exerkines in ageing and disease

Mechanistically, the preventive and therapeutic effects of physical activity can be attributed to the wide range of endogenous exercise-mobilized mediators (i.e., exerkines), which confer adaption processes across distinct tissue types. In fact, exercise is frequently promoted as a *polypill* for disease prevention and therapy,^282,283^ and the mechanisms governing these health effects are an ongoing area of research that aims to improve health outcomes in people at risk and patients.^16^ In continuation to the preclinical studies on exerkine-induced tissue adaption (see “Exerkine-induced signal transduction and biological tissue adaption”), the following paragraphs discuss the translational evidence of these mechanisms in different disease contexts. Although the clinical use of exercise-mobilized signaling molecules is not always inspired from exercise as such, we highlight the preventive and therapeutic potential of these compounds for different diseases.

#### Metabolic diseases

Preclinical studies have successfully demonstrated the therapeutic implications of different exerkines in metabolic diseases such as obesity, T2DM or fatty liver disease. As such, TGF-β2, apelin, KYNA, FGF21, and HSP72 were shown to mediate glucose-lowering and/or insulin-sensitizing effects in skeletal muscle and WAT of mice. Additionally, KYNA, irisin, lactate, and IL-6 were shown to have metabolic effects via thermogenic adaptions of WAT. For weight reduction, KYNA, lactate, IL-15, GDF15, ANGPTL4, and IL-6 were identified as exerkines with potential weight-reducing effects in obesity (Tables 2 and 3). Of note, GDF15 and lactate mediate weight reduction via central effects (i.e., suppression of appetite), while KYNA, IL-6, and ANGPTL4 have direct effects on WAT (i.e., adipose tissue lipolysis). The diverse nature of these molecular mediators suggests that exercise harnesses multiple mechanisms to exert protective effects on metabolic diseases simultaneously.

Large-scale meta-analyses of randomized controlled trials have revealed that exercise is an effective strategy for weight reduction in children, adolescents, and adults, especially when combined with dietary interventions.^284–286^ Similarly, exercise improves insulin sensitivity and glycemic control in individuals with prediabetes.^287^ In contrast, the thermogenic effects on adipose tissue of humans are a continued topic of debate. Since subcutaneous adipose tissue browning and activation of brown adipose tissue by exercise training revealed inconsistent results in humans,^288,289^ it remains questionable whether these processes depict a therapeutic avenue in metabolic diseases. Beside reduction of adipose tissue, increases in skeletal muscle mass depict a further cornerstone of weight reduction via elevated resting energy expenditure. Therefore, it is worth noting that exerkine-triggered skeletal muscle anabolism might have an additive effect in the treatment of obesity. Mechanistically, apelin, follistatin, succinate, and decorin were shown to mediate skeletal muscle hypertrophy. A similar role might be assumed for neurturin, musclin, ROS, and miRNAs, which are involved in adaptive processes of skeletal muscle tissue to exercise (Table 2 & 3). Although a potentiating effect on weight reduction might be assumed for combined aerobic and resistance exercise compared to aerobic exercise only, this is not backed by scientific evidence, as demonstrated by a similar weight reduction in obese participants performing either an isolated aerobic or a combined exercise intervention.^290^

To harness the metabolic benefits of exercise in disease context, administration of exerkine analogs (i.e., exercise mimetics) depicts a further approach. These analogs act as exerkine receptor agonists and thus induce tissue adaption in the same way as endogenously mobilized exerkines. Considering the high prevalence and disease burden of metabolic diseases,^291^ several clinical trials have applied exerkine analogs to achieve clinical improvements in premorbid individuals and patients. For instance, a clinical trial with obese participants showed insulin-sensitizing effects of apelin administration^292^ and a subsequent trial with type two diabetic patients is completed (NCT02724566), but results are not yet available. Similarly, FGF21 analogs have found their way into therapy of metabolic disorders including obesity, T2DM, and nonalcoholic fatty liver disease (for review of clinical trials see^293,294^). Clinical improvements mediated by FGF21 analogs comprise amelioration of dyslipidemia and hepatic fat content, improved fasting glucose and insulin levels, and increased high-density lipoprotein cholesterol. Thus, FGF21 is a prime example for an exercise mimetic therapy that is already applied in different diseases.

Further examples of clinical trials harnessing administration of exerkine analogs are given in Table 4. Aside from clinical trials, some studies also combined exercise mimetic approaches in healthy humans with in vitro experiments to investigate the molecular mechanisms responsible for biological tissue adaption. In an IL-6 infusion study with healthy participants, IL-6 was found to increase insulin-stimulated glucose disposal and subsequent in vitro experiments on myotubes revealed that IL-6 increases fatty acid oxidation and GLUT4-dependent glucose uptake in an AMPK-dependent manner.^295^

**Table 4:** Examples of completed and ongoing clinical trials harnessing exerkines or exerkine-induced signal transduction

Therapeutic intervention	Clinical population	Observed outcomes	Registration number and Refs	Status
Metabolic diseases				
Diabetes Mellitus type 2				
Apelin analog, 1 continuous i.v. infusion vs. placebo	Adults and older adults with T2DM, males	No results available	EudraCT 2015-004875-61	C
Sodium lactate 1 continuous i.v. infusion (600 nmol/L)	9 adults with T2DM during hypoglycemia, males and females	Lactate infusion suppresses counter regulatory hormone responses to hypoglycemia	EudraCT 2018-000684-82 NCT03730909^470^	C
rFGF21 (PEGylated) analog, 1, 5, 20 mg daily or 20 mg weekly for 12 weeks s.c. vs. placebo	130 adults with T2DM and obesity, males and females	No significant effects on HbA1c	NCT02097277^471^	C
Obesity				
Apelin analog ((pyr1)-Apelin-13), 2 doses 9 or 30 nmol/kg i.v. vs. placebo	16 overweight adult males	Improved insulin sensitivity with 30 nmol/kg	NCT02033473^292^	C
rFGF21 analog, 5, 25, 100 or 140 mg, twice weekly for 4 weeks, i.v. vs. placebo	50 adults with overweight/obesity and T2DM on a stable dose of metformin, males and females	Decrease in body weight, triglycerides, and total cholesterol with doses 100 and 140 mg.	NCT01396187^472^	C
FGF21 analog, 25, 50, 100, 150 mg infusion once weekly for 4 weeks, i.v. vs. placebo	107 adults with obesity and hypertriglyceridemia, males and females	Reduced fasting triglyceride levels with no change in weight	NCT01673178^473^	C
FGF21 analog, 3, 10 or 20 mg daily for 4 weeks, s.c. vs. placebo	46 adults with overweight/obesity and T2DM, males and females	Improvement of lipid profile with doses 10 and 20 mg, no changes in fasting glucose, insulin or weight	NCT01869959^474^	C
Cardiovascular diseases				
Heart failure				
Apelin analog, 1 dose 0.25, 2.5 or 8 µg/kg/min i.v. vs. placebo	26 adults and older adults with chronic stable heart failure, males and females	Results available, pharmacokinetic and pharmacodynamic characterization of apelin, statistical analysis pending	EudraCT 2016-001387-12 NCT02696967	C
Apelin analog, daily ascending dose (10, 30, 100 mg) for 3 weeks, p.o vs. baseline	28 adults and older adults with heart failure	Increased left ventricular ejection factor and stroke volume (MoD/volumetric assessment), no changes with Doppler method	NCT03276728^324^	C
Musculoskeletal diseases				
Rheumatoid arthritis				
Gut-selective rIL-10 3 mg daily p.o. for 12 weeks vs. placebo	Adults and older adults with active rheumatoid arthritis with inadequate (partial) response to anti-TNF therapy	No results available	EudraCT 2020-003955-14	PT
Becker Muscular Dystrophy				
3 or 6 × 10^11^ vg/kg/leg adeno-associated virus vector containing rFollistatin, 1 dose, bilateral i.m. injection	6 young adults with proof of Becker Muscular Dystrophy mutation, males	Improved walking distance and muscle histopathology (from baseline)	NCT01519349^336^	T
Neurological diseases				
Cerebral hemorrhage				
rIL-1ra 100 mg, twice daily for 3 days s.c. vs. placebo	25 adults and older adults with subacute perihematomal edema after intracerebral hemorrhage, male and female	No difference in edema extension distance	EudraCT2018-000249-38 NCT03737344^475^	C
rIL-1ra twice daily for 21 days, s.c. vs. placebo	612 adults and older adults following acute aneurysmal subarachnoid hemorrhage, males and females	No results available	EudraCT 2016-003725-42 NCT03249207	O
Cancer				
Locally advanced or metastatic cancer (various)				
IL-15 antibody fusion protein targeting CTLA-4 10–300 µg/kg, s.c.	149 adults with various histologically diagnosed unresectable, locally advanced, or metastatic tumor types, male and female	No results available	EudraCT 2022-000339-21 NCT05620134	O

*C* completed, *O* ongoing, *PT* prematurely terminated, *T* terminated, *i.m.* intramuscular, *i.v.* intravenous, *p.o.* per os, *s.c.* subcutaneous, *T2DM* type 2 diabetes mellitus, *HbA1c* glycated hemoglobin, *FGF21* fibroblast growth factor 21, *MoD* method of disk, *IL-10* Interleukin 10, *IL-1ra* Interleukin 1 receptor antagonist, *CTLA-4* cytotoxic T-lymphocyte antigen 4, *IL-15* interleukin 15, *r* recombinant

#### Cardiovascular diseases

Animal models have also linked several exerkines to structural and functional tissue adaption of the cardiovascular system. In blood vessels, preclinical studies have shown that VEGF mediates angiogenesis and collateralization of coronary arteries,^296–298^ while NO improves endothelial function.^299^ These effects relate to both, peripheral and cardiac blood vessels, and thus depict therapeutic avenues in pathologies such as hypertension, atherosclerosis or coronary heart disease. In contrast to vascular exercise adaptions, the effects on cardiac muscle tissue are more diverse: while IL-6 and VEGF induce physiological cardiac hypertrophy, HSP70 ameliorates pathological hypertrophy (Tables 2 and 3). Of note, apelin was reported to participate in both of these processes,^169^ thus suggesting preventive, and therapeutic implications of these exerkines for diseases marked by cardiac dysfunction such as heart failure. The molecular differences between physiological and pathological cardiac hypertrophy were outlined previously.^300^ Additionally, BDNF, NRG1, and HSP27 were shown to participate in cardiac repair after myocardial infarction (Tables 2 and 3).

In humans, meta-analyses have shown that exercise is an effective measure to reduce blood pressure in normotensive, pre-hypertensive, and hypertensive individuals,^301–303^ especially when combined with other lifestyle modifications such as a healthy diet.^304^ Given that exercise also reduces clinical signs of metabolic diseases (see section “Metabolic diseases”), it can be viewed as an effective strategy to prevent and/or reverse all of the clinical manifestation of metabolic syndrome, i.e., hypertension, dyslipidemia, elevated blood glucose levels, and obesity.^305^ Similarly, atherosclerosis and coronary heart disease, which share the same pathophysiology and are often a consequence of the beforementioned metabolic risk factors, are reversed by lifestyle modifications including exercise.^306^ As an early marker of atherosclerotic alterations,^307^ endothelial function has also shown to increase in response to exercise in healthy individuals and different patient collectives.^308^ Untreated coronary heart disease can ultimately result in myocardial infarction and ischemic loss of cardiac muscle tissue. However, cardiac rehabilitation involving exercise has revealed improved clinical outcomes after myocardial infarction.^309^ Ultimately, the clinical benefits of exercise also extend to pathologic alterations of the myocardium like heart failure. Despite initial doubts concerning the safety of exercise in these patient collectives, it is nowadays recognized as a safe and useful strategy to ameliorate disease burden^310,311^ and even conveys favorable effects on cardiac function.^312^ Recently, proline dehydrogenase was identified as a potential therapeutic target, since it was reduced in heart failure patients, but rescued by exercise in a rat model of heart failure.^313^

Concerning translational advances on exerkines that mediate cardiovascular adaptions, NO was repeatedly shown to trigger the protective effect of exercise on endothelial function in normotensive^259,314^ and hypertensive individuals^315^ as well as patients with coronary heart disease.^262^ Mechanistically, increased protein expression and phosphorylation of eNOS conveys these effects via an elevated production and bioavailability of NO.^259,262,314,315^ Other exerkines with implications for vascular adaptions that were translated to humans are apelin, BDNF, and VEGF. Infusion with an apelin analog resulted in peripheral and coronary vasodilation and increased cardiac output in healthy participants and patients with heart failure.^316,317^ Similarly, initial evidence suggests that BDNF might ameliorate fibrin clotting in patients with coronary heart disease.^318^ In contrast, VEGF is targeted therapeutically both to increase angiogenesis in coronary heart disease,^319^ and to decrease angiogenesis in diseases with aberrant vascularization like cancer.^320,321^ Regarding cardiac adaptions mediated by exerkines, NRG1 has yielded initial promising effects on cardiac function and hemodynamics in patients with heart failure.^322,323^ Similar cardiac benefits were found after administration of an apelin analog,^324^ thus expanding the therapeutic potential of apelin from metabolic and vascular effects (see above and section “Metabolic diseases”) to cardiac effects. Despite substantial preclinical and human evidence on the involvement of IL-6 in cardiac adaptions, translation into clinical therapy has proven difficult due to the pleiotropic effects of IL-6 and its involvement in both, cardiac protection, and cardiac pathology.^325^

#### Musculoskeletal diseases

Considering the mechanical strain that acts on the musculoskeletal system during exercise, adaption of skeletal muscle, bones, tendons, and articular cartilage to exercise training are well described. These adaptions are especially relevant against the backdrop of structural tissue decline in ageing or disease. Animal studies have successfully linked exerkines such as apelin, follistatin, succinate, or decorin to anabolic processes in skeletal muscle tissue like skeletal muscle hypertrophy. In contrast, other investigations have revealed exercise adaptions like mitochondrial biogenesis or increased oxidative capacity for exerkines such as neurturin, musclin, ROS, or miRNAs (Tables 2 and 3). Besides outlining the molecular underpinnings of exercise adaptions, these investigations hold therapeutic potential in diseases like sarcopenia, cancer cachexia, or muscular dystrophies. Similarly, osteogenic adaptions mediated by exerkines including irisin, L-BAIBA, or RCN2 have therapeutic implications in age-related and postmenopausal osteopenia and osteoporosis.

In humans, the beneficial effect of exercise in preventing or reversing these age- and disease-related alterations was repeatedly outlined by several meta-analyses. Physical activity exerts primary preventive effects on the development of sarcopenia^326^ and increases muscle mass, strength, balance, and physical performance in previously diagnosed patients.^327,328^ Some evidence suggests that similar effects are possible in cancer cachexia, with the regulation of myotube homeostasis via COP9 signalosome complex subunit 2 (COPS2) as a potential mechanism through which exercise training serves as adjuvant therapy.^329^ These results have promising implications for cancer cachexia, although the number of clinical trials in this field is sparse.^330^ Concerning skeletal effects, exercise increases bone mineral density in older adults,^331^ pre- and postmenopausal women,^332^ and patients suffering from osteopenia and osteoporosis.^333^

Due to the genetic etiology of muscular dystrophies, exercise interventions per se have yielded limited effects in these patient collectives.^334,335^ However, follistatin, an exerkine that sequesters myostatin and thus exerts anabolic effects on skeletal muscle tissue, has revealed promising results in a phase 1/2a trial on patients with Becker muscular dystrophy.^336^ A similar role for decorin might be assumed,^337^ although clinical translation is still pending. Additionally, plasma apelin levels were shown to decrease in an age-dependent manner and exercise-induced increases in apelin were associated with higher exercise performance in humans.^152^ However, therapeutic applications of apelin as an anabolic signaling molecule for skeletal muscle tissue have not been established yet. Concerning exerkines with osteogenic impact, similar results are observed in translational trials. Despite multiple indications that bone mineral density and osteogenic adaptions are related to irisin^338–340^ and L-BAIBA levels^341,342^ in humans, translation into disease therapy for conditions like osteopenia or osteoporosis has not been accomplished so far.

#### Neurological diseases

Preclinical studies on exerkines with an impact on neurological diseases have fallen into two categories. On the one hand, irisin and BDNF were shown to trigger neurogenesis with therapeutic implications in AD, vascular dementia, or Parkinson’s disease.^125–127^ On the other hand, ANGPT1 revealed angiogenic effects in an animal model of stroke, and lactate was found to participate in both, neurogenesis and angiogenesis, most likely via increased expression of VEGFA.^200,201^

In humans, exercise has proven beneficial for cognitive function of healthy older adults^343,344^ as well as people with cognitive impairment and dementia.^345^ Similarly, exercise improved cognitive function in Alzheimer’s and Parkinson’s disease specifically, with slight differences between cognitive domains.^346,347^ In stroke, exercise therapy had a positive impact on the recovery process, as mirrored by improvements in activities of daily living, and walking endurance.^348,349^ Additionally, stroke survivors that exercised 3.5–7 h or over 7 h per week had a lower relapse risk than patients that did not exercise at all.^350^ Thus, regular exercise may not only improve performance outcomes in stroke patients, but also protect from recurrence. However, despite these promising effects of exercise in stroke prevention and treatment, a more comprehensive analysis of patient-related and biological outcomes is needed to pave the way for evidence-based exercise rehabilitation in stroke survivors.^351,352^

Linking the mechanisms of neuroprotection and -recovery to the exercise-mediated health effects observed in patients has proven difficult due to the shielded position of the CNS. However, several translational trials have tried to ascertain the role of exerkines in neuronal (patho)physiology by assessing exerkine levels in human plasma or CSF. In AD and Lewy body dementia, CSF but not plasma irisin levels were reduced compared to non-demented controls. Similarly, irisin was reduced in hippocampal slices of late-stage AD patients compared to age-matched early-stage and healthy controls, suggesting that irisin levels are altered in disease marked by neuronal degeneration. A positive correlation of CSF irisin levels with age in healthy participants but not AD patients points towards similar implications of irisin.^126^ In a cross-sectional analysis of plasma irisin levels, irisin correlated with cognition in healthy individuals, but this association was lost in patients suffering from AD. Additionally, higher irisin levels were associated with advanced atrophy of several brain regions in AD patients, suggesting a compensatory role of irisin in neuronal degeneration.^353^ Of note, these results are backed by positive correlations between CSF irisin, BDNF, amyloid-beta 42 (an AD biomarker) and cognition.^354^ Similar to AD, plasma irisin levels increased in response to exercise training in Parkinson’s disease patients and a positive correlation with balance function was found.^355^ Aside from neurodegenerative diseases, ANGPT1 was repeatedly found to improve outcomes like infarction size, BBB integrity, and neurological function after stroke in rodents,^356,357^ but translation to humans is surprisingly sparse. Given that plasma ANGPT1 levels are lower in stroke patients compared to healthy controls and considering that low ANGPT1 was associated with a more severe outcome in these patients, ANGPT1-based interventions have promising therapeutic implications in humans.^358^

Since various exerkines were shown to mediate therapeutic effects in neurological diseases, a further promising approach is transfer of exercised plasma from healthy individuals to patients. This strategy offers the advantage that exercised plasma contains multiple exerkines, and might thus confer neuroprotection and symptom alleviation in a more global manner.^359,360^ Exercised plasma transfer revealed beneficial effects in a rodent AD model,^361^ and current studies such as the ExPlas study (NCT 05068830) are testing whether this approach is also feasible, safe, tolerable, and effective in AD patients.^362,363^

#### Cancer

A substantial body of mechanistic research suggests that exerkines also have therapeutic implications in cancer. In an animal study with tumor-bearing mice, exercise was shown to mobilize NK cells via β-adrenergic signaling, which then relocated to tumor tissue in an IL-6-dependent manner. Of note, the mobilization of NK cells by epinephrin and the infiltration into tumors via IL-6, yielded over 60% reduction in tumor incident and growth across different tumor models, including skin, liver, and lung models.^227^ Similar effects of exercise on anti-tumor immunity were found in pancreatic for CD8 + T cells, a further population of immune effector cells that is able to recognize and eliminate cancer cells. Exercise resulted in a strong epinephrin-dependent mobilization of CD8 + T effector cells and these immune cells required IL-15/IL-15Rα signaling to infiltrate into tumors and exert their tumorigenic function.^364^ Apart of reductions in tumor weight, IL-15Rα + CD8 + T cells also showed increased expression of PD-1 and exercise sensitized pancreatic tumors to checkpoint inhibition therapy with the most pronounced reductions in tumor wight found in exercise combined with checkpoint blockade. Ultimately, by comparing different therapy regimes, the combination of checkpoint blockade with an IL-15Rα agonist and chemotherapy yielded the highest reductions in tumor volume, thus suggesting high therapeutic potential of a triple therapy in pancreatic cancer.^364^ Besides these immunological mechanisms, there are numerous further exercise-mediated effects that suppress tumor growth such as modulation of cancer metabolism,^365^ or increased vascularization to improve hematologic delivery of cancer therapeutics.^366,367^ Comprehensive overviews of the preventive and therapeutic implications of exercise in cancer and examples of molecular mechanisms mediating these beneficial effects were outlined previously.^19,21,368,369^ However, the preclinical studies highlighted here have specifically harnessed exercise to investigate the therapeutic potential of exercise-mobilized immune effector cells such as NK cells or CD8 + T cells.

In human cancer settings, exercise has revealed remarkable results on multiple levels. Besides protecting from the development of several cancers,^370,371^ regular physical activity performed either before or after diagnosis also reduces cancer mortality.^371^ Additionally, exercise therapy has shown to improve patient-related outcomes such as side effects of chemotherapy (e.g., cancer-related fatigue)^372^ and cardiorespiratory fitness.^373^ In search of an explanation for these effects, different mechanisms are discussed, albeit caution should be devoted to the level of evidence of these findings, especially since many mechanistic insights have not been transferred to humans yet.^374^ For instance, the anti-tumor effects of exercise-mobilized immune effector cells has not been recapitulated in humans and in fact some human cancers might not be sensitive to such mechanisms in the first place due to a low tumor mutational burden and limited immunogenicity.^375,376^ Thus, although exercise-mediated effects on tumor-sensitive immune cells are a promising area of research, future translational trials will have to show how exercise shapes anti-cancer immunity in different patients collectives.

#### Ageing

The progressive deterioration of physiological integrity that characterizes ageing increases the predisposition for numerous human pathologies including cancer as well as metabolic, cardiovascular, and neurodegenerative disorders.^377^ Since ageing is a joint risk factors of these etiologically distinct diseases,^378–380^ big efforts are made to improve our understanding of the ageing process and develop therapies that enable healthy ageing. In an attempt to find common denominators of these pathologies, the “hallmarks of ageing”, formulated by López-Otín and colleagues in 2013 and updated in 2023, have provided a powerful resource that has shaped ageing research in an inimitable manner.^377,381–383^ Of note, exercise has evolved as a powerful lifestyle intervention and rejuvenation strategy that attenuates various hallmarks of ageing.^384–386^

Making use of heterochronic parabiosis, blood factors from young mice were shown to reverse age-related impairments in cognition and synaptic plasticity of old counterparts.^387^ In line with previous findings that circulating factors influence neurogenesis in an age-dependent manner,^388^ this has led to a reimagination of brain ageing as systemic event.^360,386^ Interestingly, these rejuvenating effects of young blood were reproduced in other target tissues, including skeletal muscle,^389,390^ and the vasculature,^391^ while the blood system itself (i.e., hematopoietic stem cells) remained refractory.^392^ Since exercise enhances cognitive performance and protects from age-related cognitive decline in rodents^393^ and humans,^394–396^ it was hypothesized that exercise might also confer beneficial effects on the brain via circulating factors. Indeed, plasma transfer from exercised to sedentary aged mice induced neurogenesis and cognitive improvements that were similar to those found in mice exposed to the exercise intervention.^210^

In search of the mechanistic foundation of these beneficial effects, several exerkines were discovered as molecular transducers. For instance, GPLD1, a GPI-degrading hepatokine that is enriched in plasma of healthy active elderly humans, ameliorated age-related regenerative and cognitive impairments in mice. These effects occurred downstream of GPI-anchored substrate cleavage, suggesting that enzymatic activity of GPLD1 is necessary to improve brain function.^210^ Similarly, clusterin, a complement cascade inhibitor, mediated anti-inflammatory effects on the CNS in an experimental model of brain inflammation and AD via binding to brain endothelial cells.^209^ Exercise-induced hippocampal neurogenesis was additionally shown to depend on selenium and the exercise-induced release of SEPP1. These mechanisms were effective in reversing learning deficits induced by hippocampal injury and ageing.^269^

Approaching age-related cognitive decline from a slightly different angle, platelets are increasingly moving into scientific focus for their ability to alter brain physiology.^397,398^ After discovering that exercise-activated platelets can crosstalk to the brain to induce neurogenesis,^208^ several follow-up studies have established the role of platelet factors in mediating healthy brain ageing.^205–207^ One platelet factor that has raised particular interest due to its impact on brain ageing is PF4 (also known as CXCL4). PF4 attenuates age-related neuroinflammation and improves synaptic plasticity and cognition in aged mice.^206^ Additionally, it was shown to depend on longevity factor klotho^205^ and respond to relatively short periods of exercise (1–4 days),^207^ thereby further establishing its role as neurogenic exerkine. Besides the exerkines mentioned here, there are several other mediators and additional strategies like caloric restriction with rejuvenating effects on target tissues.^359,399,400^

#### Concluding remarks

In conclusion, evidence on the translational advances of exercise-mobilized signaling molecules in disease prevention and treatment strongly depends on the progress that is made in specific diseases. For some mediators, pharmaceutical analogs have already been designed and are currently tested in clinical trials (for examples see Table 4). In these cases, modifications including PEGylation, and genetic engineering technologies are applied to increase selectivity and solubility of drugs. For optimal bioavailability, future trials will have to show which drug delivery systems are optimal for application in patients and whether further chemical or environmental modifications can improve the desired therapeutic effects.^401^ In contrast, other compounds have yielded promising results in animal models but have not found their way into clinical therapy so far. For these candidates, the translational trials presented here are often based on associations between biological parameters (e.g., plasma concentrations) and functional diseases outcomes. Although this does not causally proof that these molecules mediate preventive or therapeutic effects in humans, it demonstrates an association to the pathophysiology of different diseases. Human evidence may sometimes be limited, and we thus hope that the collection of trials presented here invites more detailed translational approaches on the preventive and therapeutic potential of exercise and the molecular mechanisms that govern exercise adaptions across multiple organ systems. Importantly, we want to point out, that although the implementation of isolated exerkine analogs into clinical therapy opens new therapeutic avenues for different diseases, exercise therapy as such is accompanied by the simultaneous release of numerous exerkines. Considering the extensive implications of these mediators in healthy tissue adaption, a stronger implementation of physical activity and exercise into disease prevention and therapy is indispensable. Additionally, it must be emphasized is that not all exercise adaptions rely on exerkines as molecular mediators. Intracellular signaling cascades are not always triggered by exerkines that act upon target cells, but might also result from other external or internal stimuli, including mechanical load,^124^ or changes in intracellular pH.^52^ Additionally, alterations in proteins, metabolites, and nucleic acids within tissues themselves might confer disease protection, without the need of systemic mobilization. For instance it was recently shown in humans that adipose tissue immune cells regulate adipocyte lipolysis via the secretion of oncostatin-M,^402^ and that this exercise-induced crosstalk does not rely on systemic mobilization of exerkines.

## Current limitations and future perspectives

As outlined in this review unraveling cause and consequence of exercise-mediated tissue adaption is essential to harness the preventive and therapeutic potential of exercise in different disease settings. It is important to note that prior health and fitness status, as well as exercise training modalities like intensity, duration, frequency, and type (e.g., endurance vs. resistance exercise) affect the secretion of exerkines and the subsequent adaption processes across different tissues.^59^ This review discussed exerkines that are secreted both, in response to acute exercise and exercise training, irrespective of the exercise modalities applied, to provide an overview of the general mechanisms through which these exerkines exert their function. However, it must be noted that most studies (especially animal models) applied endurance exercise, thus potentially biasing the results presented here.

**Fig. 5:**
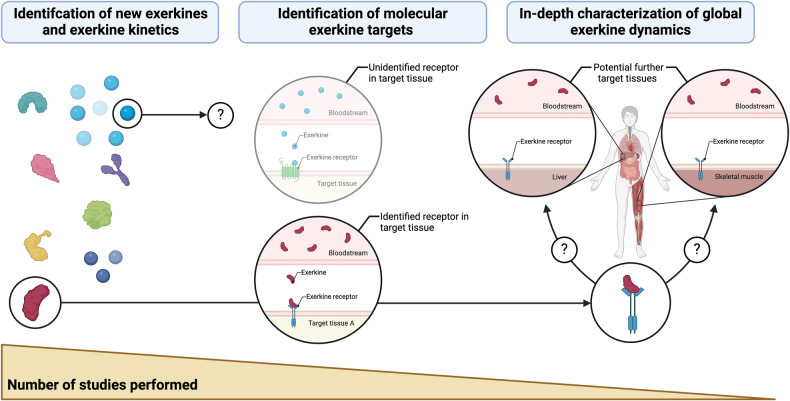
Schematic illustration of the exerkine research continuum. Owing to technological advances, remarkable progress is being made in identifying new exerkines and describing their kinetics in response to an acute bout of exercise (left section of the figure). To uncover exerkine-mediated tissue adaptions, some studies have identified molecular targets of exerkines (e.g., exerkine receptors) that transduce these effects (central section of the figure). However, less attention is devoted to potential further target tissues that also express these molecular targets. In-depth characterization of global exerkine dynamics—i.e., the interaction of exerkines with different target tissues—holds the potential to advance our understanding of the pan-tissue benefits mediated by exercise (right section of the figure). Created with BioRender.com

Exerkines form an indispensable component of healthy tissue adaption and knowledge of the molecular mechanisms harnessed by exerkines is crucial to gain a mechanistic understanding of tissue-wide adaptions in health and disease. Owing to methodological advances in untargeted and targeted mass spectrometry, metabolomic, proteomic, and lipidomic analyses boosted the discovery of new exerkines in an inimitable manner,^403^ leading to an ongoing identification of new molecules.^17,40,209,250^ Identifying the source tissue of exerkines has thus become feasible. For instance, using adeno-associated viral delivery of an engineered biotinylation enzyme, Wei and colleagues recently attributed one, in some cases even multiple, tissues of origin to many of the known and unknown exerkines.^40^ Other strategies like microdialysis-enabled sample collection of extracellular fluids depict attractive alternatives, especially in human exercise studies.^404,405^ While these technological improvements allow for detailed analyses on the one end of exerkine research, i.e., the identification of new exerkine candidates, far less investigations are situated on the other end, i.e., unraveling the detailed effects of established exerkines on target cells, specific signaling pathways, and the subsequent adaption processes in vivo (Fig. 5). Target tissues of exerkines are thus mostly identified by adopting a hypothesis-driven research strategy (e.g., using animal knockout models). One reason for this imbalance might lie in the more difficult nature of hypothesis-testing experiments compared to hypothesis-generating omics approaches. However, comprehensive tissue-wide investigations on exerkine-mediated cell signaling, and subsequent adaption processes hold high scientific potential for the pan-tissue health benefits of exercise (Fig. 5).

Given that many exerkines exert their effects on target cells in a receptor-dependent manner, a crucial question in mechanistic exercise research is whether these exerkines act on further target tissues that also express the target receptor. Mechanistically, this could explain how exercise confers health benefits across multiple tissues simultaneously. In fact, for some exerkines multiple target tissues have already been described (Table 2). To inspire future research on the health benefits of exercise across various tissues, we have summarized exercise studies that investigated exerkine receptor signaling in vivo and provide a collection of further target tissues that also express the identified exerkine receptors (Table 5). Although the mere expression of an exerkine receptor does not imply receptor activation by exerkines, the exercise-induced increase in plasma exerkine levels (in case of systemic mobilization) and the expression of exerkine receptors on potential target tissues, form the theoretical foundation for exerkine signaling and the associated tissue adaptions in vivo. To prove this, experimental verification is indispensable. Interdisciplinary initiatives like the Molecular Transducers of Physical Activity Consortium (MoTrPAC) have identified this need and are currently unraveling the molecular underpinnings of exercise-mediated health effects.^406^

**Table 5:** In vivo exercise studies investigating exerkine receptor signaling and examples of further human tissues with exerkine receptor expression

Exerkine	Exerkine receptor	Refs	Examples of further human tissues expressing exerkine receptors
Skeletal muscle			
NTN	GFRA2/ RET	^146^	GFRA2: Nervous system, bone morrow, CSF RET: PBMCs
TGF-β2	TGF-βR2	^151^	PBMCs, breast
IL-15	IL-15Rα	^149^	No data
Fractalkine	CX3CR1	^145,150^	Adrenal gland, PBMCs, liver, lungs
Musclin	NPR3	^95^	Blood, urine
Apelin	APLNR	^147,152^	Spinal chord
Cardiac muscle			
BDNF	NTRK2	^171^	CNS, CSF, PBMCs
IL-6	IL6R	^225^	Blood, nasal respiratory epithelium
VEGF	VEGFR3	^170^	Blood, breast, placenta, cardiac muscle
NRG1	ERBB2, ERBB4	^176^	ERBB2: lung, skin ERBB4: skin
White adipose tissue			
KYNA	GPR35	^185^	Blood, monocytes, neutrophils, CSF, cardiac muscle
FGF21	FGFR1/KLB	^192^	FGFR1: Blood, monocytes, neutrophils, CSF, cardiac muscle KLB: no data
Irisin	αV/β5 integrins	^183^	Integrin αV: immune cells, platelets, lymphatic tissue, fetal heart, bone marrow, bone, smooth muscle organs, lung, pancreas, prostate, reproductive organs Integrin β5: immune cells, lymphatic tissue, cerebral cortex, heart, oral epithelium, fetal gut, liver, spleen, lung, pancreas, placenta, uterus, cervix, ovary
Lactate	HCAR1	^462,463^	No data
IL-6	IL6R	^225^	Blood, nasal respiratory epithelium
FSTL1	DIP2A	^32^	No data
Bone			
Irisin	αV/β5 integrins	^183^	See above
L-BAIBA	MRGPRD	^57,194,476^	Heart
RCN2	NRP2/1 integrin	^124^	Blood, CNS, smooth muscle organs, adipocytes, amniocyte, breast, urine, skin, placenta, fetal organs
Central nervous system			
Lactate	HCAR1	^200,201,215^	No data
ANGPT1	TIE-2	^202^	Fetal heart, lung, placenta
GDF15	GFRAL/ RET	^203,477^	GFRAL: no data RET: PBMCs
Immune cells			
IL-8	IL8R	^232^	Nasal respiratory epithelium, PBMCs, monocytes
IL-6	IL6R	^224^	Blood, nasal respiratory epithelium
Succinate	SUCNR1	^51,52^	No data
Skin			
IL-15	IL-15Rα	^144^	No data
Liver			
FN1	αV/β1 integrins	^250^	Integrin αV: see above Integrin β1: blood, immune cells, platelets, lymphatic tissue, bone marrow, bone, CNS, retina, heart, smooth muscle organs, kidney, spleen, lung, adipocytes, secretory glands, prostate, skin, reproductive organs

The exerkines displayed in this table are derived from in vivo exercise studies on exerkines and exerkine receptors. Estimated protein expression was extracted from the Human Gene Database (https://www.genecards.org). By using a cutoff of 0.5 parts per million (ppm) on a base-10 log scale, we present an experimentally sound collection of target tissues that are characterized by exerkine receptor expression and therefore have the potential to transduce the signals of exerkines into tissue adaption. Of note, some of the exerkine receptors listed in the table did not meet the described criteria for inclusion as target tissue (marked with “No data”). However, this does not rule out the possibility that convincing evidence for exerkine receptor expression might originate from individual investigations *APLNR* apelin receptor, *CX3XR1* C-X3-C motif chemokine receptor 1, *IL-15Rα* Interleukin-15 receptor α, *IL-15* Interleukin 15, *TGF-βR2* transforming growth factor β receptor 2, *GFRA2* glial cell line derived neurotrophic factor family receptor α 2, *RET* REarranged during Transfection, *NTN* neurturin, *TRKB* tropomyosin-related kinase B, *BDNF* brain-derived neurotrophic factor, *NRG1* neuregulin 1, *IL-6* Interleukin 6, *IL-R* Interleukin 6 receptor, *IL-8* Interleukin 8, *IL-8R* Interleukin 8 receptor, *ERBB2/ERBB4* Erb-B2 receptor tyrosine kinase 2/Erb-B2 receptor tyrosine kinase 4, *VEGFR3* vascular endothelial growth factor receptor 3, *VEGF* vascular endothelial growth factor, *NRP2* neuropilin 2, *RCN2* reticulocalbin 2, *FSTL1* follistatin-like 1, *DIP2A* disco-interacting protein 2 homolog A, *MRGPRD* Mas-related G protein-coupled receptor type D, *L-BAIBA* β-aminoisobutyric acid, *HCAR1* hydroxycarboxylic acid receptor 1, *FGFR1* fibroblast growth factor receptor 1, *KLB* co-receptor β-Klotho, *FGF21* fibroblast growth factor 21, *GPR35* G protein-coupled receptor 35, *KYNA* kynurenic acid, *GFRAL* glial cell line derived neurotrophic factor family Receptor a Like, *GDF15* growth differentiation factor 15, *TIE-2* tyrosine-protein kinase receptor TEK, *ANGPT1* angiopoietin 1, *SUCNR1* succinate receptor 1, *PBMC* peripheral blood mononuclear cells, *CSF* cerebrospinal fluid, *CNS* central nervous system

Despite the high health-related potential of different exercise approaches, major challenges are still present as the translational value of exerkines requires detailed in vivo and in vitro knowledge on the molecular mechanism governing tissue adaption. As these might differ between healthy and diseased populations, detailed, context-dependent investigations are necessary to enable prevention of disease in healthy individuals and treatment of diseases in patients. Additionally, in dependence on the individual patient and disease characteristics, exercise modalities might be tailored to mediate the desired therapeutic effect. Considering modality-dependent differences in molecular exercise adaptions thus depicts an important topic of research for future investigations. In this context, a comprehensive overview of personalized physical activity recommendations was recently provided by Noone and colleagues.^407^ Feasibility, safety, (cost-)effectiveness, and benefit-risk assessments for different exercise therapies, including exercise mimetic approaches, and structured, well-designed randomized controlled trials with both performance and biomedical outcomes are necessary to ultimately pave the way to exercise-mediated prevention and treatment of disease.

## Conclusion

Based on the myriad of original and review articles dealing with the health effects of exerkines, this review displayed the molecular foundations of exercise therapy in disease prevention and treatment. By outlining the detailed signaling cascades triggered by exerkines, the subsequent tissue adaptions observed in vivo, and the immediate relevance of these results in the prevention and treatment of different diseases, our aim was to give a comprehensive overview of the molecular mechanisms underlying exercise therapy in disease prevention and treatment. Our special emphasis on molecular targets of exerkines and exerkine receptors as mediators of health effects originates from the observation that much progress in being made in identifying new exerkines, but far less research focuses on describing the distinct effects on potential target tissues. Indeed, cause and consequence of exercise-mediated health effects are often acquainted before the molecular target—e.g., a receptor that transduces exercise signals into tissue adaption—is identified. Precise knowledge on the tissue-specific expression patterns of exerkine receptors, however, holds the promise to improve our understanding of concomitant health effects in multiple target tissues simultaneously. Investigating the entire biological axis from exerkine kinetics over exerkine dynamics through to signal transduction and cellular adaption is challenging from different perspectives, but at least of equal importance compared to untargeted omics approaches to gain a profound understanding of how exercise mediates health benefits across our population. Exerkine kinetics and exerkine dynamics might additionally be altered by lifestyle changes, disease, or ageing (e.g., altered exerkine mobilization or exerkine receptor expression on target tissues), with crucial implications for the associated adaption processes. In disease context, several exerkines have been linked to clinical improvements in patients and some have already found their way into pharmacological therapy of different diseases. The fact that some exerkines, such as neurturin, irisin or apelin target multiple tissues via the same receptor, underlines our model of pan-tissue health effects of exerkines. Whether these multi-tissue effects also apply to other exerkine-exerkine receptor pairs remains to be elucidated.

In conclusion, we hope to shed light on exerkine-triggered signaling cascades that contribute to healthy tissue adaption, since these are of profound relevance for the prevention and treatment of various chronic diseases. Our review provides a comprehensive collection of downstream signaling cascades triggered by exerkines in vivo and fills the scientific gap created between basic mechanistic research and clinical improvements in patients by highlighting the importance of exerkine target receptors as molecular transducers of exercise-mediated health benefits. Bridging this scientific gap represents a major challenge that requires thorough consideration by the exercise medicine community to facilitate progress in the field.
